# Genome‐Wide Identification and Expression Analysis of TaDES1 Gene Family Responded to Biotic and Abiotic Stress in Wheat ( *Triticum aestivum* L.)

**DOI:** 10.1002/fsn3.70504

**Published:** 2025-07-08

**Authors:** Wenjie Kan, Yameng Gao, Ziqi Wang, Zhu Yang, Yuan Cheng, Dacheng Wang, Zhiwei Li, Caiguo Tang, Lifang Wu

**Affiliations:** ^1^ The Center for Ion Beam Bioengineering and Green Agriculture Hefei Institutes of Physical Science, Chinese Academy of Sciences Hefei Anhui China; ^2^ State Key Laboratory of Sustainable Dryland Agriculture Shanxi Agricultural University Taiyuan Shanxi China; ^3^ University of Science and Technology of China Hefei Anhui China

**Keywords:** abiotic stress, biotic stress, cysteine desulfhydrase, hydrogen sulfide, wheat

## Abstract

L‐Cysteine desulfhydrase (DES1), a key enzyme in eukaryotes, catalyzes the synthesis of hydrogen sulfide (H_2_S), which is a gaseous signaling molecule. However, the genes encoding DES1 enzymes in wheat, one of the world's most important crop species, have yet to be fully characterized. This study offers a comprehensive analysis of the wheat TaDES1 gene family and delves into its potential functions. We identified a total of 18 *TaDES1* genes, located across 13 chromosomes, categorized them into four subfamilies, and comprehensively analyzed their physicochemical properties. Furthermore, three DES1 gene families (HvDES1, OsDES1, and ZmDES1) were identified in three Poaceae species to explore the evolutionary relationships of TaDES1 and its homologs. The results indicated that segmental duplication drove the expansion of the TaDES1 family, which experienced strong purifying selection. Promoter *cis*‐elements and gene ontology (GO) enrichment analysis revealed the significant roles of this gene in the stress response, phytohormone regulation, and plant growth. miRNA target prediction analysis further explored the regulatory relationships. Transcriptomic data revealed that TaDES1 members are responsive to abiotic stresses, biotic stresses, and exogenous abscisic acid (ABA) treatment. The qPCR (RT–qPCR) results also demonstrated that the TaDES1 gene is responsive to multiple stresses. Co‐expression network analysis emphasized the importance of key *TaDES1* genes in stress responses. Finally, simple sequence repeats (SSRs) within the TaDES1 family were predicted, and variation analysis of three key *TaDES1* genes and their homologs across ten wheat cultivars was performed to explore their potential in wheat breeding.

## Graphical abstract

The TaDES1 gene family in wheat consists of 18 genes across 13 chromosomes, categorized into four subfamilies. Evolutionary analysis shows segmental duplication as a key driver of family expansion, with strong purifying selection. TaDES1 members play critical roles in stress response, phytohormone regulation, and plant growth and development, supported by promoter cis‐elements, gene ontology (GO) enrichment, transcriptomic data, RT‐qPCR, and co‐expression network studies. Simple sequence repeats (SSRs) and variation analysis further highlight their potential utility for wheat breeding.

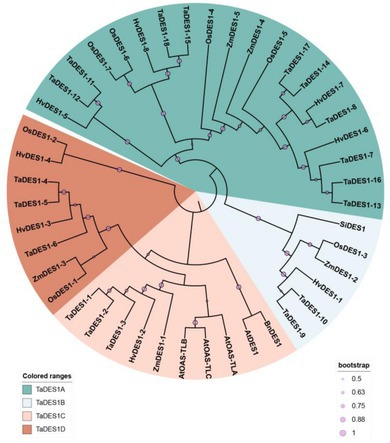

## 1 Introduction

Hydrogen sulfide (H_2_S) was initially considered a harmful gas because it is strong (Pudi et al. 2022) and has a pungent odor even at concentrations below 1 ppm. However, with the discovery of nitric oxide (NO) and carbon monoxide (CO) as endogenous signaling molecules, H_2_S has been reported as the third gasotransmitter (Huang et al. 2023). In the past few decades, H_2_S has been extensively studied in the field of mammalian medicine, with H_2_S signaling related to physiological processes in nearly all organ systems examined to date (Olson et al. 2014). Given that H_2_S has been reported to play crucial roles in cardiovascular disease treatment (Li et al. 2018), antioxidant defense (Zou et al. 2020), anti‐inflammatory responses (Gemici and Wallace 2015), cancer cell treatment (Wang et al. 2023), and antiviral activities (Pozzi et al. 2021), its potential in clinical research and therapeutic applications is immense.

Although research on H_2_S in animals has flourished, in‐depth studies of H_2_S in plants only gained momentum this century. In plants, the physiological effects of H_2_S are pervasive throughout their life cycle, from growth to development, and include the processes of seed germination (Fang et al. 2022), root development (Mukherjee and Corpas 2020), stomatal closure (Li, Liu, et al. 2024), photosynthesis in leaf tissue (Chen et al. 2011), organ abscission (Liu et al. 2020), and fruit ripening (Hu et al. 2020). Moreover, it has been observed that H_2_S exhibits a notable preservative effect in fruits like strawberries and peaches (Ziogas et al. 2018). In studies on adverse stress responses, H_2_S is recognized as playing a critical role in both biotic and abiotic stress responses. H_2_S is considered important in biotic stress responses, such as controlling Fusarium head blight (FHB) in wheat (Yao et al. 2022) and improving the photosynthetic capacity of cabbage seedlings under black rot stress (Wang et al. 2024). H_2_S also responds to various abiotic stresses, such as drought (Du et al. 2019; Li et al. 2021), salt (Li at al. 2022; Yang et al. 2023), temperature (Li, Fang, and Bai 2024), and heavy metal stresses (Luo et al. 2020).

There are three primary pathways for endogenous H_2_S synthesis in plants. The first pathway involves the direct absorption of exogenous H_2_S from the atmosphere through the leaf surface. The remaining two primary pathways for endogenous H_2_S synthesis are closely associated with cysteine (Cys) metabolism (Fang et al. 2023). Two pathways necessitate the involvement of two enzymes: *O*‐acetylserine (thiol) lyase (OAS‐TL) and Cys desulfhydrases (CDes) (Laureano‐Marín et al. 2014). The CDes family includes L‐Cys desulfhydrase (LCD, AT3G62130; Muñoz‐Vargas et al. 2023), D‐Cys desulfhydrase 1 (DCD1, AT1G48420; Bharath et al. 2012), D‐Cys desulfhydrase 2 (DCD2, AT3G26115; Bharath et al. 2012), and L‐Cys desulfhydrase 1 (DES1, AT5G28030; Alvarez et al. 2010; Zhang et al. 2021). One of the most critical clusters of these enzymes is DES1, which catalyzes the decomposition of L‐cysteine into pyruvate, ammonia, and H_2_S (Alvarez et al. 2010). *DES1* is expressed across various growth stages of plants, with particularly high expression levels observed in seedling, mature stage (Laureano‐Marín et al. 2014), and flower stages. Interestingly, DES1 was initially classified as a member of the OASTL family owing to significant sequence homology; however, compared with the authentic OAS‐TL enzyme, DES1 has substantial differences in both protein structure and function (Alvarez et al. 2010; Xie et al. 2013). DES1 and OAS‐A1 enzyme activities are important for plant cysteine homeostasis, sulfide homeostasis, and H_2_S levels (Gotor et al. 2013; Krueger et al. 2009; Romero et al. 2014).

In *Arabidopsis*, the *DES1* gene and its corresponding *des1* mutant have been extensively studied (Liu et al. 2021; Yang et al. 2023; Zhang et al. 2021; Zhang, Zhou, et al. 2020). The *AtDES1* gene is crucial for stomatal regulation in plants, photomorphogenesis, root development, delayed senescence, and resistance to drought stress, as well as in response to various growth and developmental adversities. The pathways in which AtDES1 functions include the ABA pathway (Zhang, Zhou, et al. 2020) and multiple signaling pathways involving H_2_S, NO, and hydrogen peroxide (H_2_O_2_; Liu et al. 2021; Liu and Xue 2021). In tomato ( *Solanum lycopersicum* L.), SiDES1 is believed to play a role in increasing the levels of H_2_S in response to phosphate starvation, thereby mitigating the inhibition of root growth (Fang et al. 2014). In rapeseed ( *Brassica napus* ), the *BnDES1* gene is responsive to a variety of phytohormones and may contribute to plant signal transduction processes (Xie et al. 2013).

Wheat, one of the most important and widely cultivated grain crops in the world (Erenstein et al. 2022; Zhu et al. 2025), is susceptible to the environment because of its extensive cultivation and growth characteristics (Mao et al. 2023). The increasing frequency of extreme climates exposes wheat to a variety of abiotic and biotic stresses (Abhinandan et al. 2018; He et al. 2024; Ma et al. 2021), which can severely impact yield and quality and may even lead to complete crop failure (Toreti et al. 2019). The vital role of H_2_S in the wheat stress response has been widely reported (Liu et al. 2021; Luo et al. 2020). Despite these findings, research on the critical role of H_2_S in wheat has focused predominantly on exogenous H_2_S supplementation and inhibitors/scavengers of endogenous H_2_S. Research on the endogenous synthesis of H_2_S in wheat remains limited. Zhang utilized *OsLCD* (XM015757‐751.2) for homology comparisons in the wheat genome, leading to the prediction and cloning of the TaLCD (Zhang, Liu, et al. 2020). Studies of genes homologous to *DES1* in wheat have not been reported. Given the widespread existence of DES1 as an enzyme, the lack of research in wheat is not conducive to further exploration of the impact of endogenous H_2_S on the response of wheat to stress and growth development.

Based on this background, three DES1 homologs (AtDES1, SiDES1, and BrDES1) were employed to identify wheat genome homologs. We identified 18 *TaDES1* genes across 13 chromosomes, which can be classified into four subfamilies, and studied the physicochemical properties of these family members. In addition, three DES1 gene families in Poaceae crops were identified, and the evolutionary relationships between the TaDES1 members and DES1 homologs from both Poaceae and non‐Poaceae plants were analyzed. The expansion of the TaDES1 gene family is attributed mainly to segmental duplication, with the gene family undergoing strong purifying selection. Promoter *cis*‐elements, gene ontology (GO) enrichment, miRNA target prediction, co‐expression network analysis, and transcriptome data indicate the significant potential role of this gene in phytohormone responses, light responses, stress responses, and growth and development. Based on these findings, *TaDES1‐9*, *TaDES1‐10*, and *TaDES1‐11* were identified as key members of the gene family. qPCR (RT‐qPCR) results further confirmed that TaDES1 members are responsive to abiotic and biotic stresses. Finally, simple sequence repeats (SSRs) within the TaDES1 family were predicted, and mutation analysis of three key *TaDES1* genes and their homologs across ten wheat cultivars was performed to explore their potential in wheat breeding.

## 2 Results

### 2.1 Genome‐Wide Identification of the *TaDES1* Genes in Wheat

**FIGURE 1:**
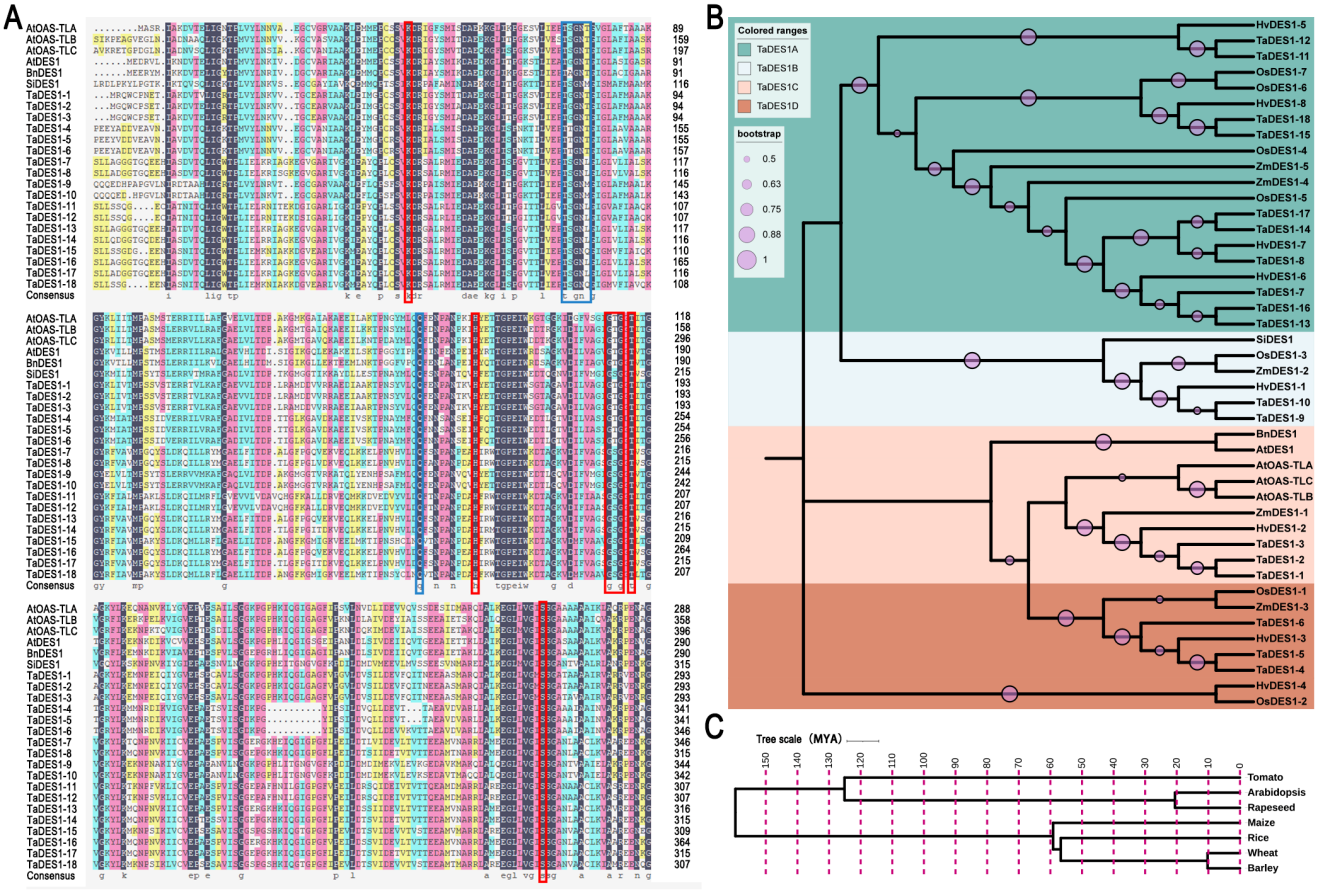
Evolutionary relationship analysis of *TaDES1* genes. (A) Amino acid sequence alignment of TaDES1 members, three DES1 homologs from three species, and three OAS‐TL isoforms in *Arabidopsis*. The PLP‐binding sites are highlighted by a red box, and the substrate‐binding site is highlighted by a blue box. (B) The phylogenetic analysis of TaDES1, HvDES1, OsDES1, and ZmDES1 gene families, along with AtDES1, BnDES1, SiDES1, AtOAS‐TLA, AtOAS‐TLB, and AtOAS‐TLC. (C) The whole‐genome evolution timescale of wheat, *Arabidopsis*, tomato, maize, rice, rapeseed, and barley.

We identified a total of 18 TaDES1 members in wheat. As shown in Figure 1A, the key distinction between DES1 and OAS‐TL lies in the presence of the non‐conservative TSGNT sequence, the non‐conservative sequence in the β8A–β9A loop of DES1, and the conserved pyridoxal 5′‐phosphate (PLP) binding site (similar to AtDES1, BnDES1, and SiDES1). All TaDES1 members presented these characteristics, indicating that they are true DES1 homologs.

The basic physicochemical properties of the TaDES1 members are detailed in Table S1. As shown in Table S1, the amino acid lengths of the TaDES1 members ranged from 327 to 396, and their molecular weights ranged from 34,593.98 to 42,244.62 Da. The theoretical isoelectric point (pI) ranged between 5.13 and 7.04, with 17 members being acidic (< 7) and only TaDES1‐9 being alkaline (> 7). The grand average hydropathicity of 9 TaDES1 members and 8 other family members was greater than 0, with one member equalling 0. The TaDES1 members presented instability indices ranging from 29.94 to 47.51, with a dichotomy in stability: half of the members were classified as stable (< 40), and the other half were classified as unstable (> 40). The aliphatic index values varied from 88.08 to 103.51 across the identified members of the TaDES1 gene family.

### 2.2 Evolutionary Relationship Analysis of *TaDES1* Genes

We identified DES1 gene families in the related Poaceae family, including barley ( *Hordeum vulgare* , HvDES1 gene family), rice ( *Oryza sativa* , OsDES1 gene family), and maize ( *Zea mays* , ZmDES1 gene family). In barley, rice, and maize, we identified 8, 7, and 5 family members, respectively, and named them according to their positions on the chromosomes. All HvDES1, OsDES1, and ZmDES1 members presented multiple characteristics of the DES1 family through amino acid sequence comparisons using DANMAN V6 (Figure S1). Notably, fewer members of the HvDES1, OsDES1, and ZmDES1 families were identified than those of the TaDES1 family. We used all members of the TaDES1, HvDES1, OsDES1, and ZmDES1 gene families together with AtDES1, BnDES1, SiDES1, AtOAS‐TLA, AtOAS‐TLB, and AtOAS‐TLC to construct a phylogenetic tree for further exploration of the evolutionary relationships (Figure 1B). According to the phylogenetic results, the TaDES1 gene family was classified into four subfamilies, namely TaDES1A, TaDES1B, TaDES1C, and TaDES1D.

The TaDES1A subfamily was the most populous, comprising ten members that collectively constitute 55.56% of the entire TaDES1 gene family. Both the TaDES1B and TaDES1C subfamilies have three members. With only two members, the TaDES1B subfamily is the smallest. The TaDES1B members cluster together with SiDES1, whereas the TaDES1C and TaDES1D members cluster together with the AtDES1 and BnDES1 families. Within the TaDES1A subfamily, only some members of the TaDES1, HvDES1, OsDES1, and ZmDES1 families are included, indicating that the DES1 from the Poaceae family may have diverged from that of cruciferous plants, and that different TaDES1 subfamily members may play different roles. Members of the TaDES1A, TaDES1B, and TaDES1C subfamilies exhibit high homology with some HvDES1, OsDES1, and ZmDES1 members. The members of the TaDES1C subfamily, as well as ZmDES1 and HvDES2, have relatively high homology with AtOAS‐TLA, AtOAS‐TLB, and AtOAS‐TLC, whereas no such phenomenon was found in the OsDES1 members. These findings suggest that the DES1 family within the Poaceae family is evolutionarily conserved but slightly different. Further analysis of the whole‐genome evolution timescale of these species revealed that the clustering relationship of TaDES1 subfamilies aligns with the overall genome evolution and species divergence timeline (Figure 1C). The species *Arabidopsis*, tomato, and rapeseed are found to have relatively close evolutionary relationships, which is in line with the relatively close evolutionary relationships among maize, rice, barley, and wheat.

### 2.3 Gene Structure, Conserved Motifs, Conserved Domain Analysis, and Protein Structure Prediction

**FIGURE 2:**
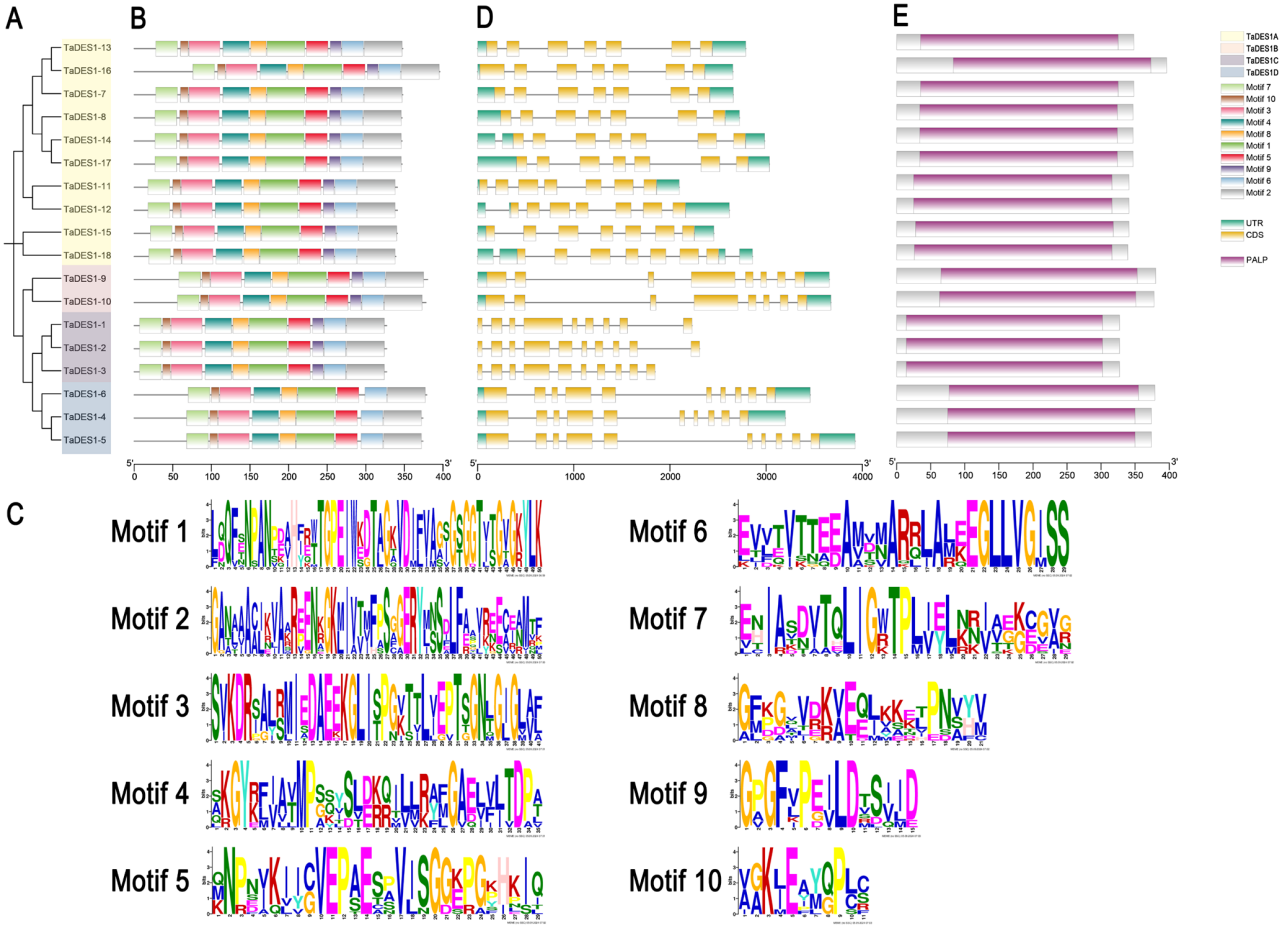
Phylogenetic relationship, conserved motifs, gene structure, and conserved domains analysis of TaDES1 gene family. (A) Phylogenetic relationship of TaDES1 members. (B) Distribution of conserved motifs among TaDES1 members. (C) Sequence logos of conserved motifs in TaDES1 members. (D) Gene structure of TaDES1 members. (E) Distribution of conserved PALP domains among TaDE1 members.

We individually constructed a phylogenetic tree of the 18 TaDES1 members to better visualize subsequent analyses (Figure 2A). MEME analysis identified 10 conserved motifs in the predicted amino acid sequences of the 18 TaDES1 proteins. All members of the TaDES1A, TaDES1B, and TaDES1C subfamilies contained all 10 motifs, whereas TaDES1D contained 9 motifs, with the exception of Motif 9 (Figure 2B). The amino acid sequences surrounding conserved amino acids are illustrated as motifs (Figure 2C). In the TaDES1A, TaDES1B, and TaDES1D subfamilies, we noted the presence of 5′ and 3′ untranslated regions (UTRs), exons, and introns as major components (Figure 2D). However, three members of the TaDES1C subfamily lacked any UTRs. The TaDES1A, TaDES1B, TaDES1C, and TaDES1D subfamilies encoded 7, 8, 9/10, and 10 exons, respectively. Notably, TaDES1‐2 and TaDES1‐3 presented the greatest number of exons, with 10 each. Additionally, as shown in Figure 2E, all members of the TaDES1 gene family possess the PALP domain.

The tertiary structure prediction results for all members of the TaDES1 family are depicted in Figure S2. Two distinct structural configurations were identified in the TaDES1A subfamily. The structural models for all TaDES1A subfamily members, except for TaDES1‐15 and TaDES1‐18, were based on templates of tryptophan synthase beta chain‐like PALP domain‐containing proteins from various species. Within the TaDES1B, TaDES1C, and TaDES1D subfamilies, the predicted tertiary structures are conserved, with templates derived from beta‐cyanoalanine synthase in soybean, cysteine synthase from *Citrullus vulgaris* , and cysteine synthase from *Brachypodium distachyon* , respectively. The tertiary structure predictions highlight the potential roles of family members in cysteine synthesis and modification. Although the protein tertiary structures within subfamilies are relatively conserved, differences exist among different subfamilies.

### 2.4 Chromosome Locations, Synteny Analysis, and Ka and Ks Calculations

**FIGURE 3:**
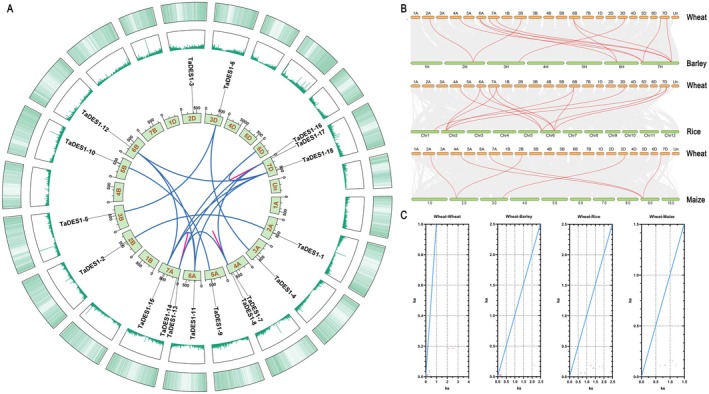
Synteny analysis of TaDES1 gene family (A) Synteny and localization analysis of *TaDES1* genes in the wheat genome. Collinear genes are highlighted with red curved lines (tandem replication) and blue curved lines (segmental duplication). The outermost two layers of the circle display the gene abundance on the chromosome. (B) Syntenic relationships of *TaDES1* genes between wheat and barley, rice, and maize. Gray lines in the background represent collinear blocks within wheat and other plant genomes. Red lines connect synteny gene pairs. (C) The Ka/Ks ratio distribution of duplicated pairs of TaDES1 members and collinear pairs between wheat and barley, rice, and maize. The blue line represents the condition where Ka/Ks = 1.

The results indicated that the 18 *TaDES1* genes were distributed heterogeneously across 13 chromosomes (Figure 3A). Notably, the A sub‐genome was found to be the most abundant among *TaDES1* genes, comprising 9 members. In contrast, the B and D sub‐genomes housed 4 and 5 members, respectively.

In our analysis of the TaDES1 gene family, significant homology among numerous members was identified, implying potential collinearity within this gene family. Consequently, we further explored the gene duplication events within the TaDES1 gene family, which include both tandem and segmental duplications. In our study, a total of 3 tandem duplication events and 15 segmental duplication events were identified among the TaDES1 gene family members (Figure 3A). To gain insights into the relationships among *DES1* genes across Poaceae species, we investigated the collinearity between wheat and its counterparts, barley, rice, and maize. As depicted in Figure 3B, *TaDES1* genes had 12, 12, and 6 syntenic gene pairs with barley, rice, and maize, respectively. Notably, the syntenic gene pairs for *TaDES1‐13*, *TaDES1‐7*, *TaDES1‐16*, and *TaDES1‐6* are present in all three species.

The nonsynonymous substitution rate (Ka), synonymous substitution rate (Ks), and Ka/Ks ratio for 18 duplicated gene pairs were determined to assess the selective pressures acting on these genes (Figure 3C). All 18 duplicated gene pairs presented Ka/Ks ratios less than 1. Additionally, all syntenic gene pairs within the DES1 gene family across the three Poaceae species also presented Ka/Ks ratios less than 1.

### 2.5 GO Analysis

**FIGURE 4:**
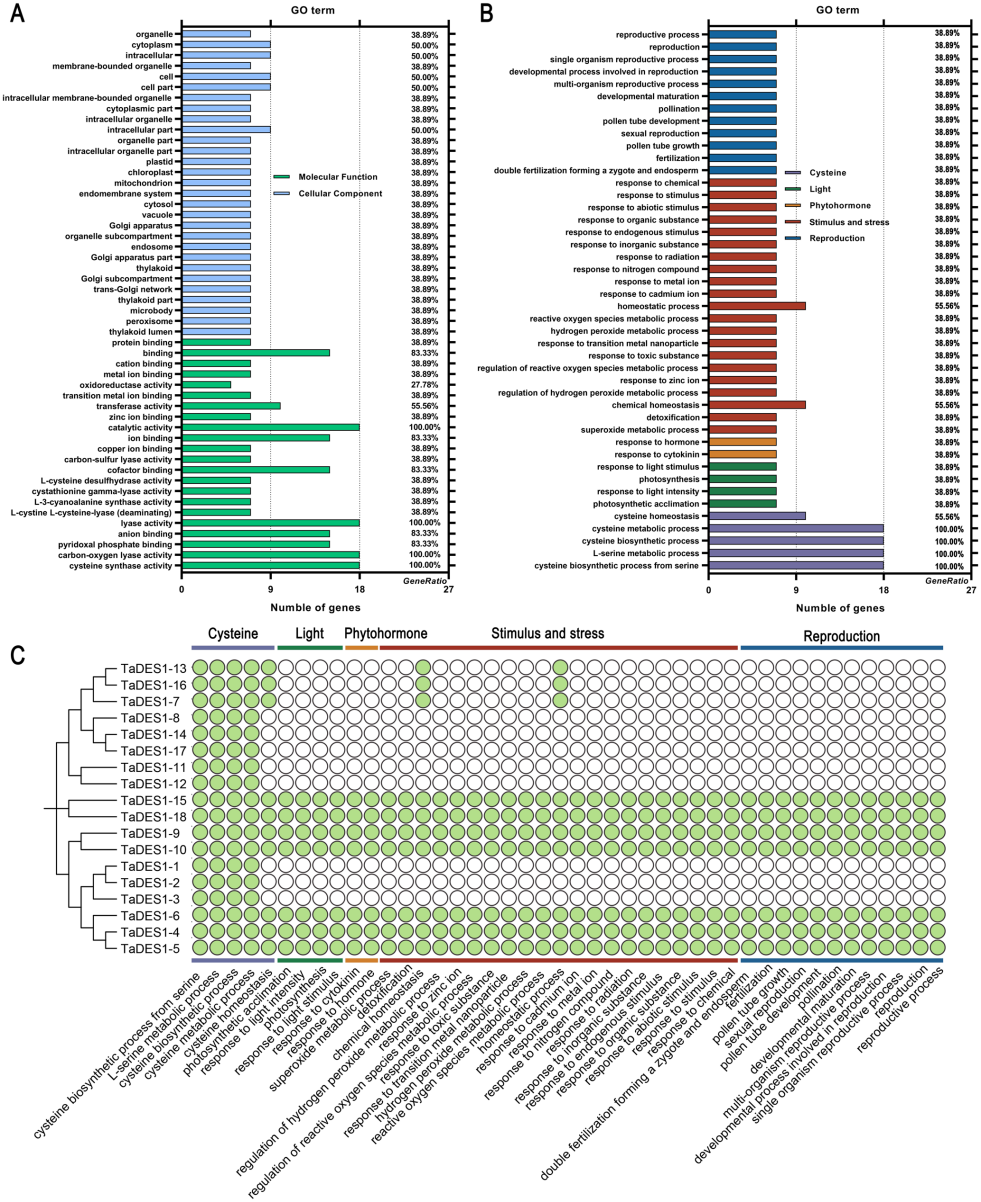
GO enrichment analysis of *TaDES*1 genes. (A) The frequency of each GO term related to molecular function and cellular component in the TaDES1 gene family members. (B) The frequency of each GO term related to light, stimulus and stress, phytohormone, reproduction, and cysteine in the TaDES1 gene family. (C) Analysis of specific GO terms related to light, stimulus and stress, phytohormone, reproduction, and cysteine in TaDES1 members, with green circles indicating the presence of these GO terms in the members.

To further predict the potential functions of theTaDES1 gene family, we conducted a GO analysis (Figure 4). A total of 180 GO terms were identified and categorized into three major classes: molecular function (22), cellular component (29), and biological process (BP; 129; Figure 4A,B). To gain a deeper understanding of TaDES1 functions, we focused on select GO terms within the BP class and classified 44 GO terms into 5 categories: light (4), stimulus and stress (21), phytohormone (2), reproduction (12), and cysteine (5). Notably, all members of the TaDES1B and TaDES1D subfamilies, as well as TaDES1–15 and TaDES1–18 from the TaDES1A subfamily, were found to encompass all the GO terms related to these five categories (Figure 4C). These results demonstrate that TaDES1 members exhibit a degree of conservation in their functional roles within their respective subfamilies. Interestingly, we observed that all family members were associated with GO terms related to cysteine synthesis and metabolism, such as cysteine biosynthetic process from serine (GO:0006535), L‐serine metabolic process (GO:0006563), cysteine biosynthetic process (GO:0019344), and cysteine metabolic process (GO:0006534).

### 2.6 Promoter *cis*‐Regulatory Element Analysis

**FIGURE 5:**
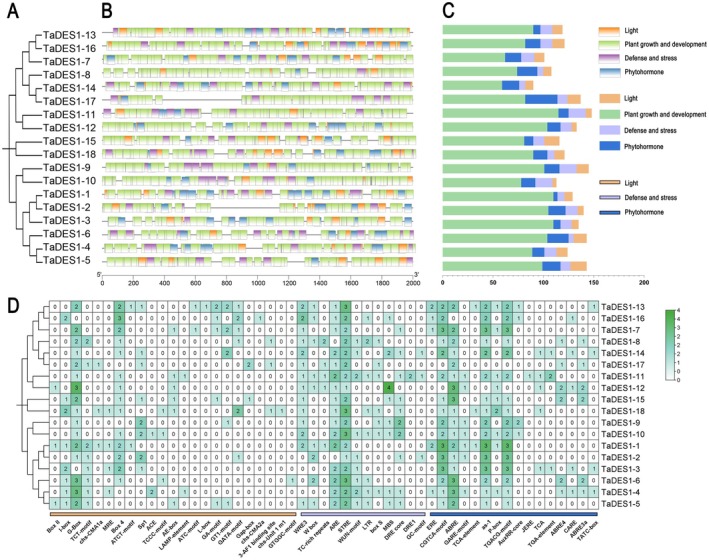
The *cis*‐regulatory element in the promoter region of *TaDES1* genes. The number represented the *cis*‐acting element numbers of *TaDES1* genes in the promoter regions.

The promoter regions located 2000 bp upstream of each *TaDES1* gene were analyzed using PlantCARE, which identified a total of 89 element types (Figure 5A,B), which were categorized into 4 groups based on their functional relevance: Plant growth and development (38 types), light (23 types), defense and stress (12 types), and phytohormone (16 types; Figure 5C). The *TaDES1* genes collectively contained 2533 elements, averaging 140.72 elements per *TaDES1* gene. Among them, the most prevalent group of promoter *cis*‐regulatory elements was the Plant growth and development group (with 1635 elements). The most abundant element type within this group was the CAAT‐box (490), with TaDES1‐10 containing the greatest number of elements (115). We further highlighted the elements of the light, defense and stress, and phytohormone groups (Figure 5D). All TaDES1 members contain light‐related elements. The most abundant types of elements in the Light group were the G‐box (48), Box4 (26), and Sp1 (13). In the phytohormone group, various phytohormone‐related elements were identified, such as auxin (IAA)‐related elements (AuxRR‐core and TGA‐element), methyl jasmonate (MeJA)‐related elements (CGTCA‐motif, JERE, TGACG‐motif), ABA‐related elements (ABRE, ABRE3a, and ABRE4), salicylic acid (SA)‐related elements (TCA‐element, as‐1, TCA), and gibberellic acid (GA)‐related elements (TATC‐box, CARE, GARE‐motif, and P‐box). In the defense and stress group, the promoter region of the TaDES1 gene family contains various elements related to low temperature (LTR), drought, and antioxidant responses. Notably, all TaDES1 members contain an STRE (stress response promoter element, AGGGG or CCCCT).

### 2.7 Prediction of Protein–Protein Interactions and miRNAs Targeting TaDES1 Members

Multiple protein–protein interaction prediction models revealed no direct interactions within the TaDES1 gene family. To further investigate their regulatory relationships, we conducted miRNA target prediction analysis to explore potential regulatory interactions at the post‐transcriptional level. Table S2 summarizes the detailed information about the predicted miRNA targeting relationships between the TaDES1 gene family and multiple miRNAs (tae‐miRxxxx) in wheat. Our analysis identified approximately 66 effective miRNA‐TaDES1 target pairs, with Expectation scores ranging from 1.5 to 5, indicating moderate confidence. Notably, pairs with scores ≤ 3 exhibit stronger biological relevance. Of particular interest, *TaDES1‐9* is the most frequently targeted gene family member, with multiple miRNAs (e.g., miR1121, miR9773, and miR5049‐3p) targeting it, suggesting that it may serve as a key miRNA‐regulated node within the family. Additionally, *TaDES1‐5*, *TaDES1‐12*, and *TaDES1‐10* also exhibit high regulatory potential, as they are targeted by several miRNAs. Notably, certain miRNAs (e.g., tae‐miR6197‐5p, tae‐miR5049‐3p, and tae‐miR1133) target multiple TaDES1 members, demonstrating redundant regulatory roles. Conversely, a single TaDES1 member can also be targeted by multiple miRNAs (e.g., *TaDES1‐9* is targeted by miR1121, miR9773, and miR5049‐3p, among others).

### 2.8 Transcriptome Data Analysis of TaDES1 Under Different Treatments

**FIGURE 6:**
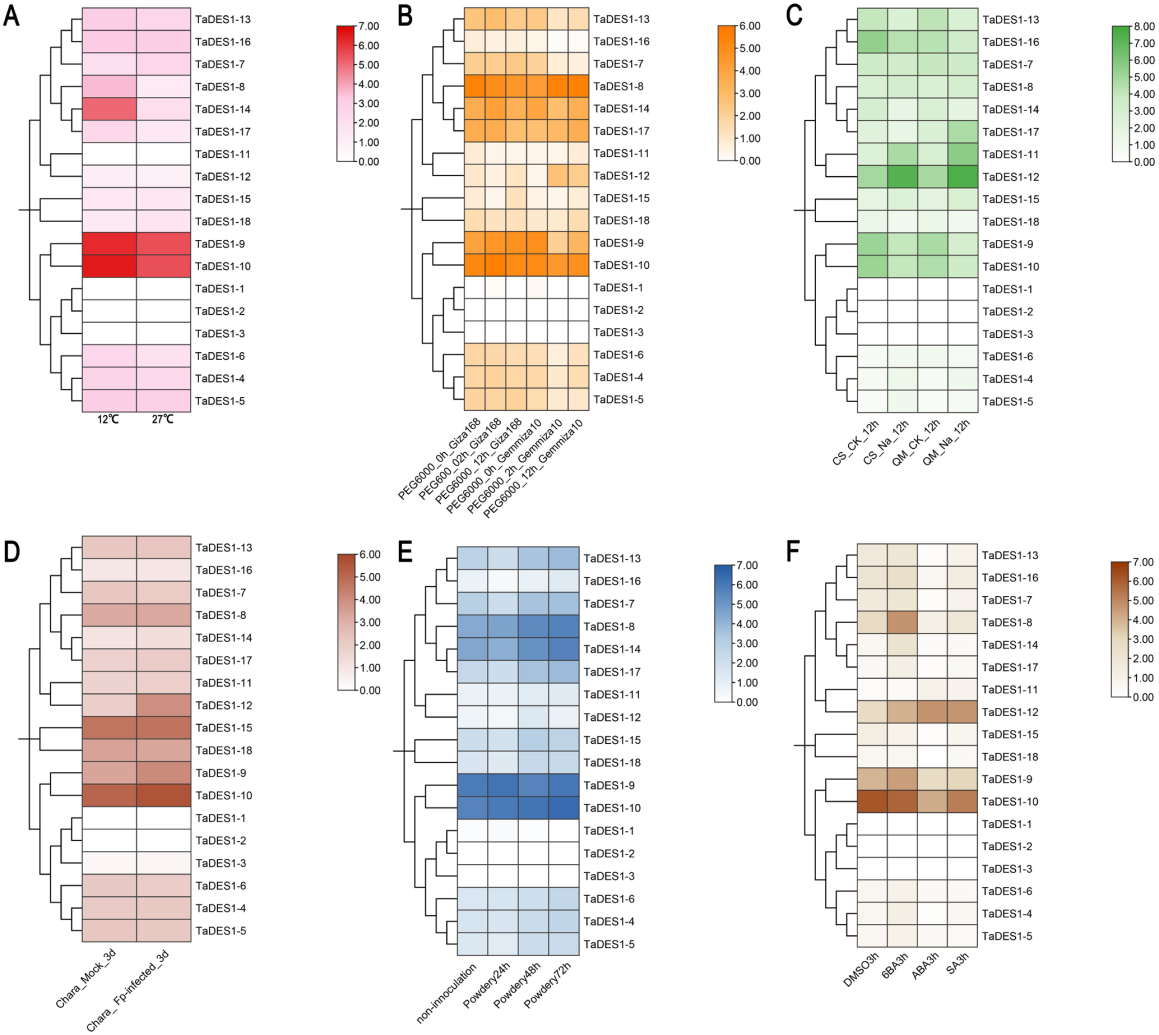
Expression analysis of the three key *TaDES1* genes under low‐temperature stress (A), drought stress (B), salt stress (C), FHB stress (D), powdery mildew stress (E), and exogenous hormone supplementation (F).

To explore the expression profiles of *TaDES1* genes under stress and phytohormone treatments, we analyzed transcriptome data from six different treatments in the WheatOmics 1.0 database. These treatments included abiotic stresses such as low temperature, drought, and salt stress; biotic stresses such as FHB and powdery mildew; and exogenous phytohormone treatments such as ABA and SA (Figure 6).

Under low‐temperature stress, the expression levels of 2 *TaDES1* genes increased, whereas 13 *TaDES1* genes presented decreased expression, and the expression of 3 members of the TaDES1C subfamily was not detected. After 14 days at 4°C, *TaDES1‐7* and *TaDES1‐11* presented 77.71% and 73.3% increases in expression, respectively (Figure 6A). In the transcriptome data analysis under drought stress, the responses of *TaDES1* genes in the drought‐tolerant wheat cultivar (Giza 168) differed from those in the drought‐sensitive wheat cultivar (Gemmiza 10; Figure 6B). After 2 h of drought stress, the two *TaDES1* genes most strongly induced by drought in Giza 168 were *TaDES1‐9* and *TaDES1‐10* whose expression levels increased by 48.10% and 39.74%, respectively, compared with their levels at 0 h. In Gemmiza 10, *TaDES‐12* and *TaDES‐8* were strongly induced by drought, with *TaDES‐12* upregulated by 1484.11% after 2 h of drought stress compared with that at 0 h. Compared with those in the control, 8 *TaDES1* genes were downregulated after 12 h of salt stress, 7 genes were upregulated, and 3 genes remained unchanged (Figure 6C). Among them, *TaDES1‐11* and *TaDES1‐12* presented strong responses to salt stress, with 470.51% and 422.68% upregulation, respectively, compared with those of the control. After wheat seedlings were immersed in *Fusarium pseudograminearum* spores for 72 h, whereas 5 *TaDES1* genes were slightly downregulated, the expression levels of the remaining 13 *TaDES1* genes were increased at 72 h compared with those of the control (Figure 6D). *TaDES1‐9*, *TaDES1‐10*, and *TaDES1‐12* were the top three upregulated genes, with increases of 76.18%, 45.10%, and 412.92%, respectively, compared with those of the control. As depicted in Figure 6E, after 72 h of treatment with powdery mildew spores, the expression levels of the other 15 *TaDES1* genes were increased, although some *TaDES1* genes showed slight downregulation or no expression. These results suggest a potential role of the TaDES1 gene family in response to biotic stress. In response to ABA and SA, the gene response patterns of the TaDES1 gene family were similar, with only *TaDES1‐11* and *TaDES1‐12* showing upregulation, *TaDES1‐2* showing no expression before and after treatment, and the expression levels of the remaining 15 *TaDES1* genes being decreased (Figure 6F).

Interestingly, three members of the TaDES1C subfamily presented low expression (or no expression) under all the treatments, whereas two members of the TaDES1B subfamily, *TaDES1‐9* and *TaDES1‐10*, showed high expression levels. Transcriptome data analysis revealed the expression patterns of the TaDES1 gene family under different stresses and treatments, highlighting the differential response patterns of the TaDES1 gene family to abiotic stress, biotic stress, and phytohormone responses, and demonstrating the complexity of stress adaptation in wheat.

### 2.9 Co‐Expression Network Analysis of the Key *TaDES1* Genes

To further investigate the functions of the key *TaDES*1 genes, we employed co‐expression analysis tools to examine genes co‐expressed with the three key *TaDES1* genes and performed GO enrichment analysis. Genes with a Pearson correlation coefficient (PCC) greater than 0.8 were identified as exhibiting strong positive correlations. Significant functional associations were observed between the key *TaDES1* genes and their co‐expressed genes.

**FIGURE 7:**
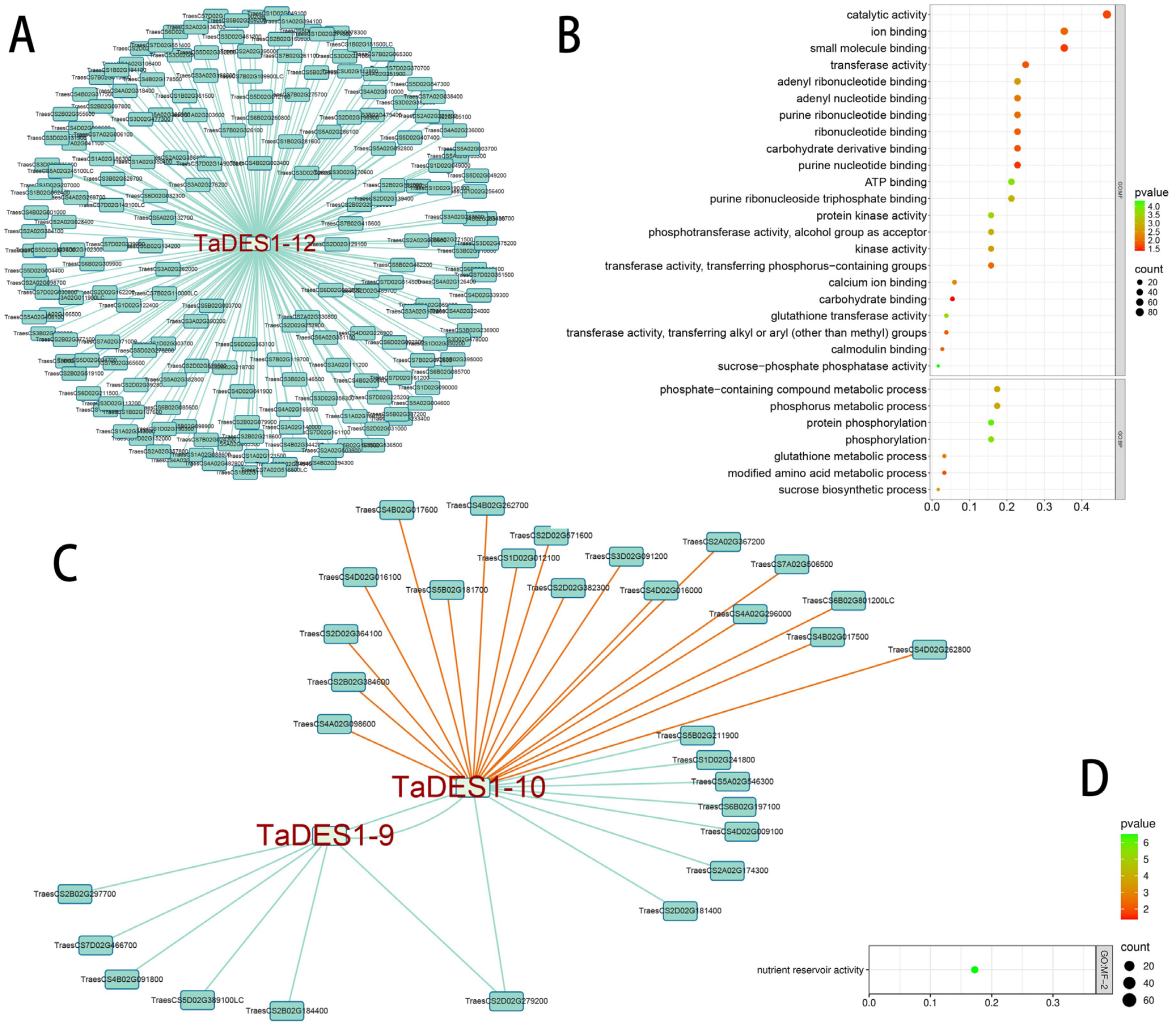
Co‐expression network analysis of the three key *TaDES1* genes. (A) Co‐expression network analysis of *TaDES1‐12*. (B) GO enrichment analysis of co‐expression genes of *TaDES1‐12*. (C) Co‐expression network analysis of *TaDES1‐9* and *TaDES1‐10*. (D) GO enrichment analysis of co‐expression genes of *TaDES1‐9* and *TaDES1‐10*.

In the Wheat Grain Database, a total of 196 genes co‐expressed with *TaDES1‐12* were identified (Figure 7A). As shown in Figure 7B, further GO analysis revealed potential roles of these genes in catalytic activity, transferase activity, metabolic processes (particularly phosphorylation compound metabolism), signal transduction, and the stress response. Notably, catalytic activity (86 genes) was the most significant category, suggesting that the TaDES1 gene family is closely associated with genes involved in enzymatic reactions. In the Wheat Multiple Tissues Database, 34 genes co‐expressed with *TaDES1‐9* and *TaDES1‐10* were identified (Figure 7C). Interestingly, several negative correlations of TaDES1‐10 were observed and *TaDES1‐10* was found to have 18 negatively co‐expressed genes. Notably, *TaDES1‐9* and *TaDES1‐10* are co‐expressed. GO analysis of the co‐expressed genes of *TaDES1‐9* and *TaDES1‐10* revealed significant enrichment in the “nutrient reservoir activity” functional category (Figure 7D).

### 2.10 Analysis of the Three Key *TaDES1* Genes Expression Under Various Different Stress Treatments

**FIGURE 8:**
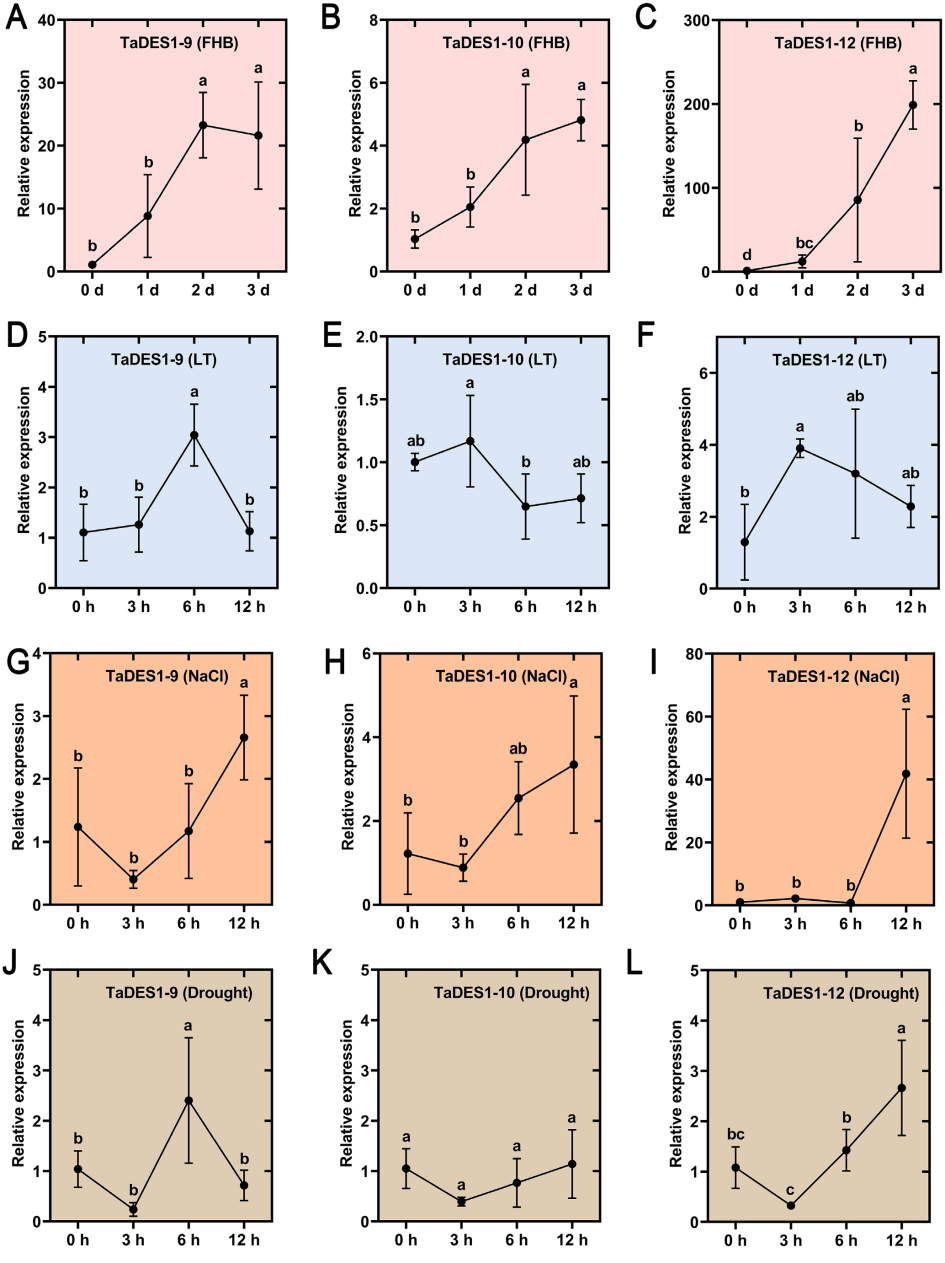
Expression analysis of the three key *TaDES1* genes under four stress treatments.

Based on the aforementioned studies, *TaDES1‐9*, *TaDES1‐10*, and *TaDES1‐12* are likely key members of the TaDES1 gene family involved in resistance to abiotic and biotic stress. To investigate whether these key TaDES1 members respond to biotic and abiotic stresses, we examined the expression patterns of *TaDES1‐9*, *TaDES1‐10*, and *TaDES1‐12* under FHB, low temperature, salt, and drought stresses (Figure 8). Following inoculation with FHB spores, *TaDES1‐9*, *TaDES1‐10*, and *TaDES1‐12* presented strong expression, with *TaDES1‐9* reaching its highest relative expression level at 2 days, which was 21.73 times greater than that at 0 days (Figure 8A). Both *TaDES1‐10* and *TaDES1‐12* presented similar expression patterns, with their expression levels gradually increasing over the three days following FHB inoculation (Figure 8B,C). Notably, *TaDES1‐12* exhibited a significant 191.02‐fold increase at 3 days, highlighting its potential critical role in stress resistance (Figure 8C). Under low‐temperature stress, all three genes presented similar expression patterns, with their relative expression initially increasing but then decreasing within 12 h post‐stress (Figure 8D–F). *TaDES1‐9*, *TaDES1‐10*, and *TaDES1‐12* reached their peak expression levels at 6, 3, and 3 h post low‐temperature stress, respectively. Under salt stress, all three genes presented the highest relative expression at 12 h, which was significantly greater than that at 0 h (Figure 8G–I). Under drought stress, *TaDES1‐9* reached its peak expression at 6 h poststress (Figure 8J), whereas *TaDES1‐10* and *TaDES1‐12* exhibited similar expression patterns to those under salt stress, with their highest expression at 12 h (Figure 8K,L).

### 2.11 SSRs Analysis of TaDES1 Members

Among the TaDES1 members identified, SSRs were detected only in *TaDES1‐2*, *TaDES1‐8*, *TaDES1‐10*, *TaDES1‐14*, and *TaDES1‐17*. Table S3 provides detailed information about these SSR markers, including their identifiers, types, sequences, sizes, start positions, and end positions. The positions of all SSR markers are distributed across different genomic regions. All the predicted SSR markers belong to the “p1” type, characterized by single‐nucleotide repeat units, with adenine (A) and thymine (T) as the predominant repeat nucleotides. These p1‐type SSRs, which are typically indicative of mononucleotide repeats, are commonly found in plant genomes because of their stability and potential for high polymorphism. The number of repeat units in the SSR markers varies, ranging from 10 to 13. For instance, the SSR marker for *TaDES1‐10* consists of 10 adenine (A) monomers, whereas the SSR marker for TaDES1‐14 contains 13 thymine (T) monomers.

### 2.12 Variation Analysis of Three Key *TaDES1* Genes and Their Homologs Across Nine Wheat Cultivars

The analysis of the three key *TaDES1* genes and their homologs across ten Chinese wheat cultivars revealed a high degree of sequence conservation (Table S4), with identity percentages ranging from 99.11% to 99.95% (Table S5, Figure S3). The variations in the *TaDES1* genes and their homologs were exclusively due to substitutions. *TaDES1‐9* and *TaDES1‐10* had fewer substitution sites, with two and three sites, respectively. TaDES1‐12 and its homologs exhibited two distinct sequence types, differing at twelve sites. Further analysis of the amino acid sequences of all the members revealed that the amino acid sequences of TaDES1‐9 and its homologs (Figure S4), which had the fewest substitution sites, remained unchanged. In contrast, three substitution sites in *TaDES1‐10* led to three amino acid changes, and the two distinct amino acid sequences of *TaDES1‐12* differed at four sites.

## 3 Discussion

Wheat, as one of the most crucial staple crops worldwide, is facing potential threats to its yield due to the increasing frequency of extreme weather events and other environmental pressures (Heino et al. 2023). In recent years, H_2_S has been widely reported in studies related to plant stress responses (Liu et al. 2021), and its endogenous synthesis pathways in plants have also received increasing attention. DES1, a pivotal enzyme in endogenous H_2_S biosynthesis, was initially identified in *Arabidopsis* (Alvarez et al. 2010). It is now recognized for its significant roles in plant growth and response to environmental stressors (Alvarez et al. 2010; Liu and Xue 2021; Xie et al. 2013; Zhang et al. 2021). Currently, there have been no reports on the presence of DES1 in Poaceae species such as wheat.

In this comprehensive study, we identified 18 members of the TaDES1 gene family in wheat using bioinformatics approaches. Using comparable bioinformatics strategies, we also identified the corresponding DES1 gene families in barley, rice, and maize. The authenticity of the TaDES1 members, including their homologs HvDES1, OsDES1, and ZmDES1, is supported by structural features analysis. DES1 was previously considered an isoform of OAS‐TL because of the high similarity in its primary structure, and both contain all the conserved residues responsible for PLP‐binding (Alvarez et al. 2010; Xie et al. 2013), a finding that is corroborated by our research (Figures 1 and S1). Multiple studies have indicated that DES1 is not just an isoform of OAS‐TL, but rather a homologue. For example, DES1 has a significantly higher affinity for l‐Cys as a substrate, with its affinity being more than 10 times greater than that of OAS‐TL (Alvarez et al. 2010). The amino acid sequence TSGNT, which is highly conserved in OAS‐TL, is notably conserved in DES1 (Alvarez et al. 2010; Xie et al. 2013). Similarly, DES1 contains non‐conserved amino acid substitutions in the β8A–β9A loop, a region that is highly conserved among genuine OAS‐TL enzymes (Alvarez et al. 2010; Xie et al. 2013). Phylogenetic analyses of AtDES1, BrDES1, and SiDES1, as well as the members of TaDES1, HvDES1, OsDES1, and ZmDES1, have classified the TaDES1 gene family into four distinct subfamilies: TaDES1A, TaDES1B, TaDES1C, and TaDES1D. Comprehensive analyses of gene structure, conserved motifs, domain conservation, and protein structure prediction have revealed that TaDES1 members are conserved within their respective subfamilies. Unlike the other three subfamilies, members of the TaDES1D subfamily do not contain Motif 9, and members of the TaDES1C subfamily lack any UTRs. These findings may indicate functional divergence among members of the subfamily during the evolutionary process. Interestingly, the domain conservation prediction of the *TaDES1* genes revealed that all the family members contained the PALP domain, providing further evidence for the conservative function of the DES1 gene family members in binding with PLP (Alvarez et al. 2010; Xie et al. 2013).

The TaDES1 gene family comprises 18 members, which is greater than those in the HvDES1 (8), OsDES1 (7), and ZmDES1 (7) gene families. Most higher plants have undergone polyploidization, a critical event in the evolution and shaping of plant genomes (Moghe and Shiu 2014) This disparity may be attributed to the hexaploid nature of wheat, with two rounds of allopolyploidization events contributing to its larger genome (Naxin et al. 2018). Furthermore, the expansion of the TaDES1 gene family in wheat may be attributed to diverse environmental conditions and potential stress risks, which require increased endogenous H_2_S production. Based on this hypothesis, we further investigated the occurrence of gene family expansion, prompting an exploration of gene duplication events. A total of 18 gene duplication events were identified, with a significantly greater number of segmental duplication events (15) than tandem duplication events (3), indicating a potential role for segmental duplication in the expansion of the TaDES1 gene family. Segmental duplication is a prevalent mechanism for the expansion of gene families across diverse plant species (Khan et al. 2020). Further Ka/Ks calculations were conducted. All 18 duplicated gene pairs presented Ka/Ks ratios less than 1, indicating strong purifying selection acting on the *TaDES1* genes over their evolutionary history (Wang, Yan, et al. 2022). The *TaDES1* genes had 12, 12, and 6 syntenic gene pairs with barley, rice, and maize, respectively. The lower number of syntenic gene pairs with maize might be attributed to the more distant phylogenetic relationship between wheat and maize. All syntenic gene pairs within the DES1 gene family across the three Poaceae species presented Ka/Ks ratios less than 1, indicating the predominant role of purifying selection in eliminating deleterious mutations and maintaining protein sequence conservation during the evolution of DES1 in these Poaceae species.

In the GO analysis, the BP class can be divided into 5 categories: light, stimulus, and stress, phytohormone, reproduction, and cysteine. All family members were found to be associated with GO terms related to cysteine synthesis and metabolism, indicating the potentially important roles of the family members in cysteine homeostasis (Álvarez et al. 2012). Cysteine contributes to the establishment and signaling of plant responses to pathogens, either directly or indirectly (Álvarez et al. 2012; Romero et al. 2014). The role of H_2_S, a byproduct of cysteine catalyzed by DES1, has been extensively reported to play multifaceted roles in photosynthesis (Chen et al. 2011; Liu et al. 2021; Yasir et al. 2023). H_2_S can mitigate various stresses in plants by enhancing photosynthetic processes (Chen et al. 2016, 2011; Li, Gao, et al. 2015; Younis and Mansour 2024). Interestingly, other hydrogen sulfide synthesis‐related enzymes, including LCD, have also been implicated to be involved in the light signaling network and photosynthesis (Zhang, Liu, et al. 2020; Zhang, Zhou, et al. 2020). These findings suggest that the roles of H_2_S synthesis‐related enzymes in photosynthesis and the light signaling network are extensive and conserved. In studies of phytohormones, ABA‐activated DES1 produces H_2_S in guard cells, thereby contributing to haem oxygenase‐modulated stomatal closure (Shen et al. 2020; Zhang, Liu, et al. 2020; Zhang, Zhou, et al. 2020). Moreover, Me‐JA (Su et al. 2023), SA (Pan et al. 2020), and other phytohormones are reportedly involved in the regulation of H_2_S, suggesting that the key enzyme for hydrogen sulfide synthesis, DES1, may also play a potential role in these processes (Laureano‐Marín et al. 2014). In *Arabidopsis*, the *DES1* gene is considered to play a significant role during the reproductive stage (Laureano‐Marín et al. 2014). In another study, H_2_S modulated plant flowering by altering the alternative splicing pattern of flowering‐related genes (Ma et al. 2024). In addition to the TaDES1B subfamily members, all the TaDES1 members presented GO terms related to stimulus and stress, suggesting their roles in the stress response.

To predict the functions of TaDES1 members, we analyzed the promoter *cis*‐regulatory elements. Diverse external signals trigger the activation of inducible promoters and engage specific *cis*‐acting elements within promoter regions (Kabir et al. 2022). Similar to the results of the GO analysis, the analysis of *cis*‐acting elements in the promoter regions revealed the potential functions of the TaDES1 gene family in terms of plant growth and development, phytohormones, the light response, and the stress response. Several phytohormone‐, drought‐, light‐, and growth‐related elements, including regulatory elements involved in flowering and motifs related to leaf maturation and senescence, were also detected in the promoter region of the *DES1* gene in *Arabidopsis* (Laureano‐Marín et al. 2014). These findings are consistent with the results of our study. The *TaDES1* genes contain various stress‐related elements, such as low temperature‐related elements (LTR) and drought‐related elements. The key role of *DES1* in drought stress has been widely reported (Shen et al. 2020; Zhang, Liu, et al. 2020; Zhang, Zhou, et al. 2020). The role of H_2_S in mitigating low‐temperature stress in plants has also been widely reported (Li, Fang, and Bai 2024; Wang, Chen, et al. 2022; Wang, Zhao, et al. 2022), however, research on the response of the DES1 to low‐temperature stress remains limited, with only a few studies available, such as the study on cucumber *DES1* genes (Liu et al. 2019).

The absence of predicted protein–protein interactions within the TaDES1 gene family may stem from several factors. Prediction tools might not capture plant‐specific or post‐translationally modified interactions (Ramazi and Zahiri 2021). Additionally, the lack of identified interactions might reflect the limited experimental data on TaDES1 interactions (Pan et al. 2021). Context‐dependent interactions occurring under specific environmental conditions or in particular tissues could also evade general prediction models (Rizzetto et al. 2018). TaDES1 proteins can function primarily as monomers or interact with non‐homologous proteins, which current prediction methods may miss. Despite the lack of direct protein–protein interactions among TaDES1 family members, our miRNA target prediction identified 66 effective miRNA–TaDES1 target pairs, suggesting a significant role for miRNAs in modulating the expression of these genes. Among the TaDES1 family members, *TaDES1‐9* emerged as a key miRNA‐regulated node that is targeted by multiple miRNAs. This finding further underscores the central role of *TaDES1‐9* within the family and suggests that it may serve as a critical hub for miRNA‐mediated regulation. Similarly, another potential key *TaDES1* gene, *TaDES1‐10*, has significant regulatory potential, further highlighting its critical role within the TaDES1 gene family.

To further validate the function of TaDES1 members, we conducted transcriptome analysis under six different treatments, including abiotic stresses such as low temperature, drought, and salt stress; biotic stresses such as powdery mildew and FHB; and exogenous hormone treatments with ABA and SA. Under low‐temperature stress, *TaDES1‐7* and *TaDES1‐11*, whose expression levels were relatively high, both contain low‐temperature response elements in their promoter regions. Under simulated drought stress with PEG6000, TaDES1 members from drought‐tolerant or drought‐sensitive wheat cultivars presented two distinct expression patterns. This may be attributed to the different genetic backgrounds and genotype variations. The expression profiles of ABA‐related genes in drought‐tolerant or drought‐sensitive wheat cultivars significantly differed under drought stress (Cevher‐Keskin et al. 2023), and DES1 is closely linked to the ABA signaling pathway (Shen et al. 2020; Zhou et al. 2021). In response to salt stress, members of the TaDES1B subfamily were strongly induced, suggesting potential key roles for *TaDES1‐11* and *TaDES1‐1*2 in the salt stress response. Interestingly, in the transcriptome analysis of the ABA and SA treatment groups, *TaDES1‐11* and *TaDES1‐12* also presented significantly increased expression levels, making them the only two members of the entire gene family whose expression levels were increased. Transcriptome data from powdery mildew and FHB infections revealed the crucial roles of the TaDES1 gene family in biotic stress responses. Fumigation with exogenous H_2_S significantly alleviated FHB in wheat seedlings (Yao et al. 2022). Exogenous H_2_S not only inhibited the growth of *F. graminearum* (Zhu et al. 2023) but also enhanced the antioxidant capacity of wheat seedlings (Yao et al. 2022). Interestingly, we observed a significant commonality in the transcriptome data, where all three members of the TaDES1C subfamily presented consistently low expression levels before and after the six treatments (with some showing zero expression levels). In contrast, members of the TaDES1B subfamily presented higher expression levels than did other family members across all the treatments. These results indicate a certain level of conservation of function within subfamilies while also suggesting functional divergence within the TaDES1 subfamily during evolution. Based on the above analysis, we posit that *TaDES1‐9*, *TaDES1‐10*, and *TaDES1‐12* are key functional members of the family. Subsequent qPCR validation confirmed their roles in response to abiotic stresses, including low temperature, drought, salt stress, and biotic stresses, such as FHB, which is consistent with previous findings. Moreover, the roles of these three key genes in the stress response suggest that they could serve as promising candidate genes for improving wheat resistance to environmental stresses in the future.

Co‐expression network analysis further validated the pivotal roles of the TaDES1 gene family in stress adaptation. The analysis of these genes co‐expressed with *TaDES1‐9*, *TaDES1‐10*, and *TaDES1‐12* provides valuable insights into their biological roles and molecular functions. The genes co‐expressed with *TaDES1‐9* and *TaDES1‐10* were enriched in the “nutrient storage activity” category, which aligns with the potential role of TaDES1 in plant metabolism, particularly in the management of nutrient reserves under varying environmental conditions. The negative correlation of *TaDES1‐10* suggests a possible antagonistic or tissue‐specific expression pattern, warranting further investigation to determine whether these correlations indicate antagonistic functions or regulatory mechanisms within the TaDES1 gene family. The strong co‐expression relationship between *TaDES1‐12* and 198 other genes suggests a central role for *TaDES1‐12* in gene expression regulation, with significant functional associations with other genes. The GO analysis of these co‐expressed genes suggested that, in addition to its crucial catalytic role as an endogenous hydrogen sulfide synthase, *TaDES1‐12* likely plays key roles in stress responses, metabolic regulation, and signaling. The functions related to phosphatase and kinase activities are particularly noteworthy. The regulatory functions indicated by kinase and phosphatase activities further suggest that *TaDES1‐12* could be a pivotal player in signaling networks that integrate metabolic cues with developmental or stress‐related responses. Kinase and phosphatase activities play crucial roles in the H_2_S‐mediated stress response signaling pathway in plants (Liu et al. 2021; Chen et al. 2020). Additionally, the associations with stress‐related processes, such as glutathione metabolism and calcium ion binding, further underscore the potential of *TaDES1‐12* in regulating plant responses to both abiotic and biotic stresses (Kushwaha and Singh 2020; Liu et al. 2021).

To further investigate the potential of the TaDES1 gene family in breeding, we performed the prediction of SSRs in the TaDES1 gene family and variation analysis of three key *TaDES1* genes and their homologs across ten wheat cultivars. The identification of gene‐specific SSR markers within the TaDES1 family provides valuable insights into their genetic diversity and potential regulatory roles. Our analysis identified multiple SSR loci across the TaDES1 gene family with a particular focus on the key member *TaDES1‐10*, which plays a critical role within this gene family. These findings suggest that SSRs may play functional roles in modulating gene stability and regulatory interactions within the TaDES1 gene family. The observed frequency of mononucleotide SSRs further highlights their potential utility as molecular markers for studying genetic variation and evolutionary dynamics in wheat. Future studies leveraging these SSRs could enhance our understanding of the functional diversification and adaptive evolution of the TaDES1 gene family. The variation analysis of three key *TaDES1* genes and their homologs across ten wheat cultivars revealed that the types of variations were conserved, with only substitutions being observed. Additionally, the *TaDES1* genes and their homologs had only two types of sequences, further highlighting the conservation of variations. The high sequence identity observed in most of the genes underscores their essential roles in wheat development and environmental adaptation. The complete identity of the amino acid sequences of *TaDES1‐9* reflects the conserved function of this gene in cultivated wheat. In contrast, *TaDES1‐10*, *TaDES1‐12*, and their respective homologs exhibit two distinct types of amino acid sequences. These variations in the *TaDES1* genes could influence the response of wheat to environmental stresses, such as drought or salinity, highlighting their potential for use in breeding programs aimed at improving stress tolerance and productivity in wheat. Future research should focus on whether the functions of homologous genes in different cultivars are consistent to further clarify the roles of these genes.

## 4 Materials and Methods

### 4.1 Plant Materials

The wheat variety “Chinese Spring” used in this experiment was harvested from the experimental field of Zhongke Taihe Experimental Station in 2023. The wheat seeds were selected and placed in a clean beaker with a 5% NaClO solution for cleaning for 5 min and then rinsed six times with pure water. The wheat seeds were then placed in a clean Petri dish with five layers of filter paper, supplemented with appropriate pure water, and placed in the dark for germination. After 24 h, the wheat seeds were synchronized in a freezer at 4°C. Wheat seedlings with uniform growth were selected 24 h later for further experiments.

Abiotic stress treatment: Wheat seedlings were grown to one leaf and one heart in a plant growth chamber with 1/4 strength Hoagland nutrient solution, and then subjected to drought stress, salt stress, and low‐temperature stress. Drought stress was simulated with a 20% PEG6000 solution; salt stress was simulated with a 200 mmol·L^−1^ NaCl solution; and low‐temperature stress was simulated at 4°C. Samples were collected from wheat seedling leaves at 0, 3, 6, and 12 h under each treatment. Biological stress treatment: The *Fusarium graminearum* inoculation method followed the procedure described by Yao et al. (2022). Two days post‐sowing (dps), the top 1–2 mm of the coleoptiles was removed, and 2 μL of *Fusarium graminearum* inoculum was applied to the remaining seedlings. Samples were collected from wheat seedling leaves at 0, 1, 2, and 3 days after inoculation.

### 4.2 Gene Identification, Sequence Retrieval, and Naming of TaDES1 Members in Wheat

The genome sequence of wheat was downloaded from the Ensembl Plants database (http://plants.ensembl.org/index.html). A BLASTP method with a threshold of 1e^−5^ was employed to search for TaDES1 members in the genome sequence of wheat using the amino acid sequences of three DES1 homologs (AtDES1: AED93766.1; SiDES1: XP_004248049.1; BrDES1: AFS17242.1 L) from *Arabidopsis* (Alvarez et al. 2010), tomato (Fang et al. 2014), and rapeseed (Xie et al. 2013). A key feature of DES1 is the replacement of some amino acid residues in the highly conserved sequence TSGNT (Alvarez et al. 2010; Xie et al. 2013). Additionally, DES1 exhibits a non‐conservative amino acid sequence in the β8A–β9A loop, a region that is highly conserved among authentic OASTL enzymes (Alvarez et al. 2010; Xie et al. 2013). Furthermore, DES1 retains the conservation of all residues critical for pyridoxal 5′‐phosphate (PLP) binding (Xie et al. 2013). Based on the description of DES1, TaDES1 candidates that did not meet the criteria were excluded. The identified TaDES1 candidates were compared with 3 DES1 homologs including AtDES1, BnDES1, and SiDES1, as well as the three members of the OAS‐TL family (AtOAS‐TLA: NP_001190733.1; AtOAS‐TLB: NP_181903.1; AtOAS‐TLC: NP_191535.2) using DNAMAN V6. To further validate the presence of the PF00291 domain (PALP: Pyridoxal‐phosphate dependent enzyme), all members of the TaDES1 gene family were confirmed for the domain using the online tool InterPro (https://www.ebi.ac.uk/interpro/) and the protein sequence vs. the profile‐HMM database (https://www.ebi.ac.uk/Tools/hmmer/search/hmmscan). The remaining sequences after eliminating redundancies were considered as TaDES1 members. The amino acid length, molecular weight (MW), isoelectric point (pI), and other physicochemical properties of the TaDES1 members were determined using the ExPASy server (www.expasy.org).

### 4.3 Evolutionary Relationship Analysis of TaDES1 Members

We used the same method described above to identify DES1 family members in three Poaceae plants, barley, rice, and maize, three Poaceae plants. Following strict screening, we identified three gene families, HvDES1, OsDES1, and ZmDES1. The obtained family members were subsequently subjected to sequence alignment analysis using DNAMAN V6, and the online tool InterPro (https://www.ebi.ac.uk/interpro/) was subsequently used to confirm the presence of the PALP domain. The genomic data and annotations for the barley, rice, and maize genomes were obtained from Ensembl Plants (https://plants.ensembl.org/info/data/ftp/index.html). We used all members of TaDES1, HvDES1, OsDES1, and ZmDES1, as well as AtDES1, BnDES1, SiDES1, AtOAS‐TLA, AtOAS‐TLB, and AtOAS‐TLC to construct the phylogenetic tree. The phylogenetic tree was constructed using the neighbor‐joining method with 1000 bootstrap replicates in MEGA11 software. The evolutionary time scales covering wheat, barley, rice, maize, *Arabidopsis*, oilseed rape, and tomato were obtained from the TimeTree website (http://timetree.org/). The online tool iTOL V6 (https://itol.embl.de/) was used to annotate and display the evolutionary relationships.

### 4.4 Gene Structure, Conserved Motifs, Conserved Domain Analysis, and Protein Structure Prediction

The Gene Structure View (Advanced) feature in TBtools II was used to analyze and visualize the evolutionary relationships, gene structures, conserved motifs, and domains of TaDES1 members. Gene sets for *Triticum aestivum* can be retrieved from the Ensembl Plants database, accessible via their FTP site (https://plants.ensembl.org/info/data/ftp/index.html). The evolutionary relationships among family members can be analyzed using the Neighbor‐joining Tree constructed with MEGA 11, which also provides insights into their phylogenetic relationships. The motif distribution and sequence logos of motifs in TaDES1 members can be determined using the MEME suite, which is available on the MEME website (https://meme‐suite.org/meme/tools/meme). In addition, the distribution of PALP within the TaDES1 members can be investigated using the NCBI Web CD search tool, available at (https://www.ncbi.nlm.nih.gov/Structure/bwrpsb/bwrpsb.cgi). Swiss‐Model (https://swissmodel.expasy.org/) was used to predict advanced protein structure.

### 4.5 Chromosome Locations, Synteny Analysis, and Ka and Ks Calculation

The chromosome locations of *TaDES1* genes were obtained from genome annotation data, with each *TaDES1* gene mapped to its corresponding locus in the wheat genome and visualized using TBtools II. The wheat genome library and GFF file were used for MCScanX for synteny analysis with setting the standard to 1e^−5^ and BlastHits number of 5 in TBtools II. The Advanced Circos feature in TBtools II was utilized for further synteny analysis and visualization. Syntenic relationships of *TaDES1* genes between wheat and barley, rice, and maize were analyzed using the One Step MCScanX feature in TBtools II. For each duplicated gene pair, the Simple Ka/Ks Calculator (NG) feature in TBtools II was used to calculate the values of Ks, Ka, and Ka/Ks.

### 4.6 GO Analysis

GO analysis of TaDES1 proteins was analyzed using the agriGO V2 (http://bioinfo.cau.edu.cn/agriGO/index.php), then displayed by GraphPad Prism 9 and TBtools II.

### 4.7 Promoter *Cis*‐Regulatory Element Analysis of TaDES1 Members

Utilizing the GTF/GFF3 sequence extractor function of TBtools II, we extracted the upstream 2000 bp sequences of TaDES1 gene family members from wheat genome data. Subsequently, the extracted sequences were submitted to the PlantCARE website (http://bioinformatics.psb.ugent.be/webtools/plantcare/html/) for *cis*‐regulatory element prediction, followed by visualization using GraphPhad Prism 9 and TBtools II.

### 4.8 Prediction of Protein–Protein Interactions and miRNAs Targeting TaDES1 Members

The STRING (Version: 12.0) databases (https://string‐db.org/), WheatCENet (http://bioinformatics.cau.edu.cn/WheatCENet/index.php), and WheatOmics 1.0 (http://wheatomics.sdau.edu.cn/wheatPPI/index.html) were used to identify the protein–protein interactions. The prediction analysis of miRNAs targeting the TaDES1 members was performed using psRNATarget (https://www.zhaolab.org/psRNATarget/analysis?function=1), with the default parameters provided by the website.

### 4.9 Transcriptome Data Analysis of TaDES1 in Different Treatments

The transcriptome data of the TaDES1 gene family were retrieved from the WheatOmics 1.0 database (http://wheatomics.sdau.edu.cn/). Abiotic stress transcriptome data included drought (Abulfaraj 2023), salt (Zhang et al. 2016), and low temperature (Li, Zheng, et al. 2015), while biotic stress data included FHB (Powell et al. 2017) and powdery mildew (Zhang et al. 2014). Exogenous phytohormones data processing involved 6BA, ABA, and SA (Chen et al. 2020). Subsequently, the downloaded data were analyzed and visualized using TBtools II.

### 4.10 Co‐Expression Network Analysis of the Key *TaDES1* Genes

Co‐expression network analysis of three key *TaDES1* genes was performed using the WheatOmics 1.0 database (http://wheatomics.sdau.edu.cn/coexpression/index.html) with a threshold of PCC ≥ 0.8. The co‐expression data for *TaDES1‐9* and *TaDES1‐10* were retrieved from the Wheat Grain database, and data for *TaDES1‐12* were sourced from the Wheat Multiple Tissues database. Visualization was carried out using Cytoscape. Furthermore, GO analysis was conducted on the co‐expressed genes of these *TaDES1* genes, employing the methodology outlined in previous studies. Visualization of the GO enrichment was performed using the online tool Enrichment Dot Bubble. Additionally, GO analysis was performed on the co‐expressed genes of these *TaDES1* genes using g:Profiler (https://biit.cs.ut.ee/gprofiler/gost). Visualization of the GO enrichment was performed using the online tool Enrichment Dot Bubble (https://www.bioinformatics.com.cn/).

### 4.11 RNA Extraction and qRT‐PCR Analysis

RNA extraction and qRT‐PCR analysis were performed by Shanghai Biozeron Biotechnology CO., LTD. The primer sequences used for qRT‐PCR are listed in Table S6.

### 4.12 SSR Analysis of the TaDES1 Members

SSRs in the TaDES1 members were identified using the MISA‐web tool (https://webblast.ipk‐gatersleben.de/misa/), with the default parameters provided by the website.

### 4.13 Variation Analysis of Three Key *TaDES1* Genes and Their Homologs Across Nine Wheat Cultivars

To identify significant genetic diversity and potential allelic variation, we performed sequence alignment of three key *TaDES1* genes (*TaDES1‐9*, *TaDES1‐10*, and *TaDES1‐12*) and their homologue in ten Chinese wheat cultivars (CM42, AMN, BJ8, ZM22, XN6028, MZM, NC4, XY6, and YM158). The sequences of these genes were obtained from the WheatOmics 1.0 database (http://wheatomics.sdau.edu.cn). The alignment focused on identifying substitutions, insertions, and deletions in the CDSs of these genes. Variations were annotated using DNAMAN software, and the identity percentages were calculated to assess sequence conservation among the cultivars.

## Author Contributions

**Wenjie Kan:** conceptualization (equal), data curation (equal), formal analysis (equal), funding acquisition (equal), investigation (equal), methodology (equal), project administration (equal), writing – original draft (equal). **Yameng Gao:** conceptualization (equal), data curation (equal), formal analysis (equal). **Ziqi Wang:** conceptualization (equal), data curation (equal), investigation (equal). **Zhu Yang:** formal analysis (equal). **Yuan Cheng:** data curation (equal), formal analysis (equal). **Dacheng Wang:** formal analysis (equal). **Zhiwei Li:** investigation (equal). **Caiguo Tang:** conceptualization (equal), formal analysis (equal). **Lifang Wu:** conceptualization (equal), resources (equal), supervision (equal), writing – review and editing (equal).

## Conflicts of Interest

The authors declare no conflicts of interest.

## Supporting information


**Figure S1.** Amino acid sequence alignment of ZmDES1, HvDES1, and OsDES1 members, three DES1 homologs from three species, and three OAS‐TL isoforms in *Arabidopsis*. The PLP‐binding sites are highlighted by a red box, and the substrate‐binding site is highlighted by a blue box.
**Figure S2.** The tertiary structure of the TaDES1 members.
**Figure S3.** Alignment with three key *TaDES1* genes and their homologs across 10 wheat cultivars.
**Figure S4.** Alignment with the amino acid sequences of three key TaDES1 members and their homologs across 10 wheat cultivars.

**Table S1.** Physicochemical properties of the TaDES1 members.
**Table S2.** Predicted miRNA targets in the TaDES1 members.
**Table S3.** Predicted gene‐specific SSR markers in genomic sequences of TaDES1 members.
**Table S4.** The name of the three key *TaDES1* genes and their homologs across 10 wheat cultivars.
**Table S5.** Variations in three key *TaDES1* genes and their homologs across 10 wheat cultivars.
**Table S6.** Primer sequences used for qPCR.

**Data S1.** Deduced amino acid sequences of DES1 analyzed in this study.

## Data Availability

The datasets generated for this study are available in online repositories. Plant genome data used in this study is available through the Ensemble Plants database (https://plants.ensembl.org/index.html), with Taxonomy IDs for wheat (4565), *Arabidopsis* (3702), tomato (4081), rapeseed (3708), maize (4577), rice (39947), and barley (112509). Transcriptome data was obtained from the WheatOmics 1.0 database (http://wheatomics.sdau.edu.cn/expression/index.html).
