# Supplementary material for: Genome‐Wide Identification and Expression Analysis of TaDES1 Gene Family Responded to Biotic and Abiotic Stress in Wheat ( Triticum aestivum L.)
Source: Food Sci Nutr. 2025 Jul 8;13(7):e70504. doi: 10.1002/fsn3.70504 (PMC12235670; doi:10.1002/fsn3.70504)
Supplement: Supplementary file 1 — Figure S1. Amino acid sequence alignment of ZmDES1, HvDES1, and OsDES1 members, three DES1 homologs from three species, and three OAS‐TL isoforms in Arabidopsis. The PLP‐binding sites are highlighted by a red box, and the substrate‐binding site is highlighted by a blue box. Figure S2. The tertiary structure of the TaDES1 members. Figure S3. Alignment with three key TaDES1 genes and their homologs across 10 wheat cultivars. Figure S4. Alignment with the amino acid sequences of three key TaDES1 members and their homologs across 10 wheat cultivars. [file FSN3-13-e70504-s002.docx]

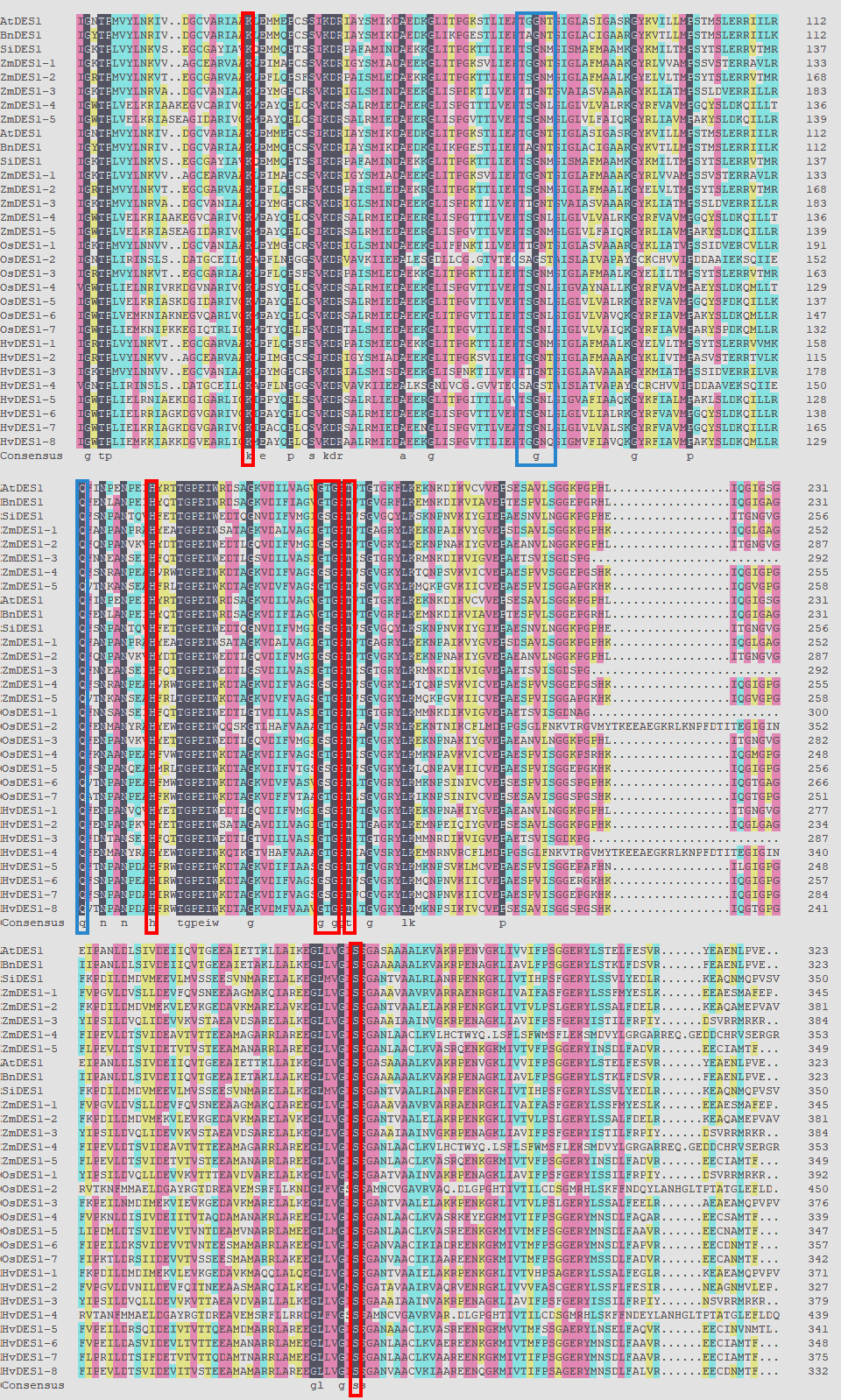


**Supplementary Figure S1** Amino acid sequence alignment of ZmDES1, HvDES1, and OsDES1 members, three DES1 homologs from three species, and three OAS-TL isoforms in *Arabidopsis*. The PLP-binding sites are highlighted by a red box, and the substrate-binding site is highlighted by a blue box.


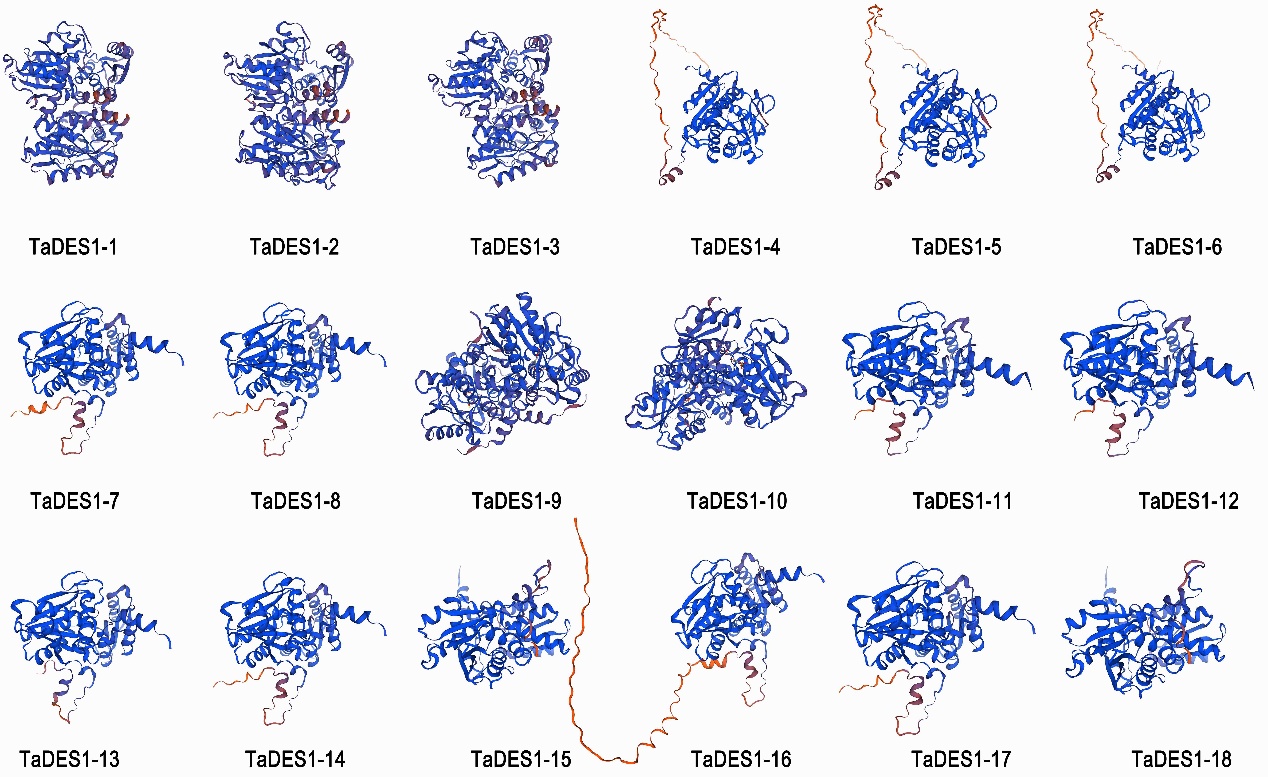


**Supplementary Figure S2** The tertiary structure of the TaDES1 members.


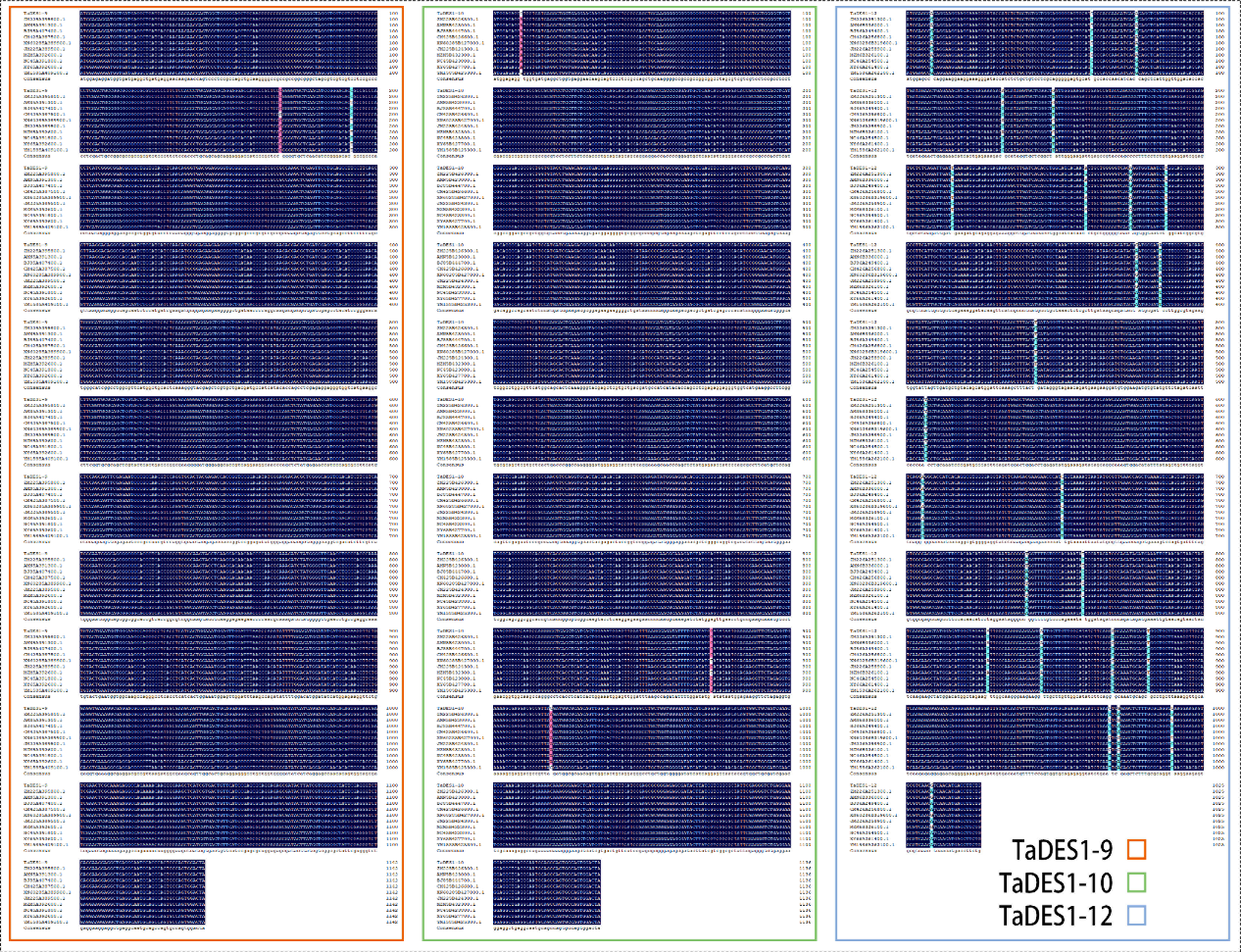


**Supplementary Figure S3** Alignment with three key *TaDES1* genes and their homologs across ten wheat cultivars.


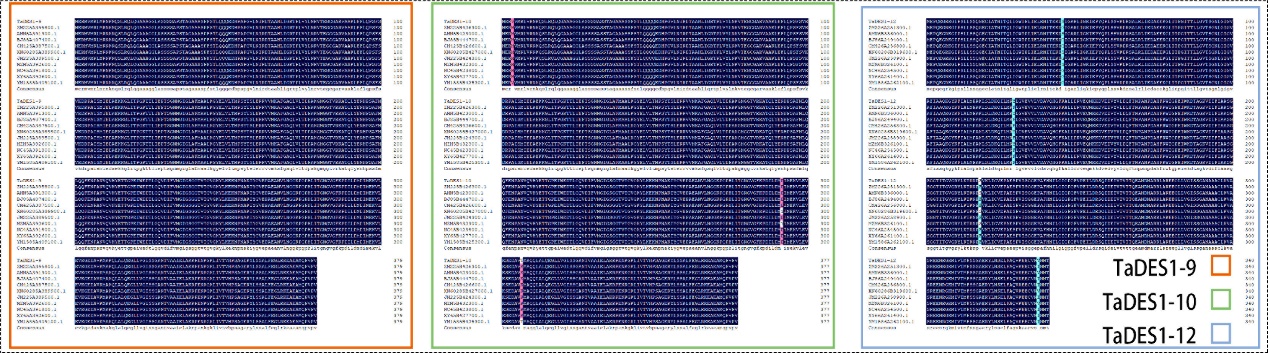


**Supplementary Figure S4** Alignment with the amino acid sequences of three key TaDES1 members and their homologs across ten wheat cultivars.
