# Supplementary material for: Genome‐Wide Identification and Expression Analysis of TaDES1 Gene Family Responded to Biotic and Abiotic Stress in Wheat ( Triticum aestivum L.)
Source: Food Sci Nutr. 2025 Jul 8;13(7):e70504. doi: 10.1002/fsn3.70504 (PMC12235670; doi:10.1002/fsn3.70504)
Supplement: Supplementary file 2 — Table S1. Physicochemical properties of the TaDES1 members. Table S2. Predicted miRNA targets in the TaDES1 members. Table S3. Predicted gene‐specific SSR markers in genomic sequences of TaDES1 members. Table S4. The name of the three key TaDES1 genes and their homologs across 10 wheat cultivars. Table S5. Variations in three key TaDES1 genes and their homologs across 10 wheat cultivars. Table S6. Primer sequences used for qPCR. [file FSN3-13-e70504-s001.docx]

| **Physicochemical properties of the TaDES1 members.** | | | | | | |
| --- | --- | --- | --- | --- | --- | --- |
| **Sequence ID** | **Number of Amino Acid** | **Molecular Weight** | **Theoretical pI** | **Instability Index** | **Aliphatic Index** | **Grand Average of Hydropathicity** |
| **TaDES1-1** | 327 | 34720.18 | 5.91 | 29.94 | 96.64 | 0.101 |
| **TaDES1-2** | 327 | 34593.98 | 5.51 | 32.09 | 95.75 | 0.105 |
| **TaDES1-3** | 327 | 34681.95 | 5.37 | 31.97 | 95.14 | 0.097 |
| **TaDES1-4** | 374 | 39702.97 | 5.9 | 40.24 | 102.3 | 0.164 |
| **TaDES1-5** | 374 | 39692.97 | 6.24 | 38.69 | 101.76 | 0.176 |
| **TaDES1-6** | 379 | 40171.67 | 6.77 | 39.94 | 103.51 | 0.204 |
| **TaDES1-7** | 348 | 36993.61 | 5.13 | 44.78 | 96.95 | 0.007 |
| **TaDES1-8** | 347 | 36835.38 | 5.16 | 41.04 | 94.41 | -0.015 |
| **TaDES1-9** | 380 | 40451.61 | 7.04 | 36.55 | 88.08 | -0.131 |
| **TaDES1-10** | 378 | 40188.14 | 6.35 | 35.74 | 89.81 | -0.106 |
| **TaDES1-11** | 341 | 36988.75 | 5.37 | 44.38 | 100.38 | 0.025 |
| **TaDES1-12** | 341 | 36960.65 | 5.37 | 43.69 | 100.09 | 0 |
| **TaDES1-13** | 348 | 36994.71 | 5.29 | 45.39 | 96.38 | 0.01 |
| **TaDES1-14** | 347 | 36871.39 | 5.16 | 41.03 | 93.83 | -0.027 |
| **TaDES1-15** | 341 | 36554.3 | 5.28 | 37.81 | 90.94 | -0.012 |
| **TaDES1-16** | 396 | 42244.62 | 5.9 | 47.51 | 92.6 | -0.092 |
| **TaDES1-17** | 347 | 36867.38 | 5.16 | 40.77 | 93.29 | -0.034 |
| **TaDES1-18** | 339 | 36441.24 | 5.4 | 37.47 | 89.73 | -0.011 |

**Supplementary Table S1** Physicochemical properties of the TaDES1 members.

**Supplementary Table S2** Predicted miRNA targets in the TaDES1 members.

| **miRNA_Acc.** | **Target_Acc.** | **Expectation** | **UPE$** | **miRNA_start** | **miRNA_end** | **Target_start** | **Target_end** | **miRNA_aligned_fragment** | **alignment** | **Target_aligned_fragment** | **Inhibition** | **Multiplicity** |
| --- | --- | --- | --- | --- | --- | --- | --- | --- | --- | --- | --- | --- |
| tae-miR1121 | TaDES1-15 | 1.5 | -1 | 1 | 22 | 1174 | 1195 | AGUAGUGAUCUAAACGCUCUUA | ::::: ::::::::::::::.: | UAAGAACGUUUAGAUCACUAUU | Cleavage | 1 |
| tae-miR1121 | TaDES1-9 | 2.5 | -1 | 1 | 22 | 1984 | 2005 | AGUAGUGAUCUAAACGCUCUUA | ::::::.::::: :::::::.: | UAAGAGUGUUUACAUCACUAUU | Translation | 1 |
| tae-miR6197-5p | TaDES1-9 | 3 | -1 | 1 | 21 | 2020 | 2040 | UCUGUAAACAAAUGUAGGACG | ::.::.::: ::::::.:: | GCUCUUAUAUUAGUUUACGGA | Translation | 1 |
| tae-miR9675-3p | TaDES1-10 | 3 | -1 | 1 | 21 | 2360 | 2380 | UUUAUGAUCACUCUCGUUUUG | :.:.: :::.:::.::::.:: | CGAGAGGAGGGUGGUCAUGAA | Cleavage | 1 |
| tae-miR9675-3p | TaDES1-9 | 3 | -1 | 1 | 21 | 2331 | 2351 | UUUAUGAUCACUCUCGUUUUG | :.:.: :::.:::.::::.:: | CGAGAGGAGGGUGGUCAUGAA | Cleavage | 1 |
| tae-miR9773 | TaDES1-16 | 3 | -1 | 1 | 24 | 56 | 79 | UUUGUUUUUAUGUUAUUUUGUGAA | ::::: ::.: ::::::::: | GCUCCAAAAAAAUACAAAAACAAA | Translation | 1 |
| tae-miR1133 | TaDES1-12 | 4 | -1 | 1 | 22 | 2450 | 2471 | CAUAUACUCCCUCCGUCCGAAA | :: .: ::::::::::::: : | UUGUGAACGGAGGGAGUAUCUU | Cleavage | 1 |
| tae-miR6197-5p | TaDES1-15 | 4 | -1 | 1 | 21 | 1210 | 1230 | UCUGUAAACAAAUGUAGGACG | ::.::.::: :::::::: | UCUCUUAUAUUAAUUUACAGA | Translation | 1 |
| tae-miR1120a | TaDES1-9 | 4.5 | -1 | 1 | 24 | 1969 | 1992 | ACAUUCUUAUAUUAUGAGACGGAG | :::.:: ::::::::::.::: | CUCUGUAAACUAAUAUAAGAGUGU | Cleavage | 1 |
| tae-miR1120c-5p | TaDES1-9 | 4.5 | -1 | 1 | 21 | 2011 | 2031 | UAAUAUAAGAACGUUUUUGAC | ::::: :::::::::: | GAUCUAAACGCUCUUAUAUUA | Translation | 1 |
| tae-miR1130a | TaDES1-12 | 4.5 | -1 | 1 | 23 | 2441 | 2462 | CCUCCGUCUCGUAAUGUAAGACG | : :::::.:::..:: ::::::: | CAUCUUAUAUUGUGA-ACGGAGG | Cleavage | 1 |
| tae-miR1130a | TaDES1-2 | 4.5 | -1 | 1 | 23 | 842 | 864 | CCUCCGUCUCGUAAUGUAAGACG | :.:::::::::::: :: | CCAGGUGCAUUACGAGACGACGG | Cleavage | 1 |
| tae-miR1130a | TaDES1-1 | 4.5 | -1 | 1 | 23 | 742 | 764 | CCUCCGUCUCGUAAUGUAAGACG | :.:::::::::::: :: | GAAGGUGCAUUACGAGACGACGG | Cleavage | 1 |
| tae-miR5085 | TaDES1-2 | 4.5 | -1 | 1 | 21 | 1871 | 1891 | AAGGACAUUUUUUGUGGCCUG | ..: :.: :::.:::::.:: | UGGACUAGAAAGAAUGUUCUC | Cleavage | 1 |
| tae-miR6197-5p | TaDES1-14 | 4.5 | -1 | 1 | 21 | 1933 | 1952 | UCUGUAAACAAAUGUAGGACG | ::.::.::::::: .:::: | UAUCUUAUAUUUGUU-GCAGA | Cleavage | 1 |
| tae-miR6201 | TaDES1-12 | 4.5 | -1 | 1 | 22 | 1478 | 1499 | UGACCCUGAGGCACUCAUACCG | ::: . :::.::::: ::: | AUUUAUAGCUGCUUCAGGUUCA | Cleavage | 1 |
| tae-miR6201 | TaDES1-11 | 4.5 | -1 | 1 | 22 | 1167 | 1188 | UGACCCUGAGGCACUCAUACCG | ::: . :::.::::: ::: | AUUUAUAGCUGCUUCAGGUUCA | Cleavage | 1 |
| tae-miR9657b-3p | TaDES1-5 | 4.5 | -1 | 1 | 21 | 2265 | 2285 | CGUGCUUCCUCGUCGAACGGU | : ::.::.:::: .:::.: | GACCUUUGAUGAGGUGGCAUG | Cleavage | 2 |
| tae-miR9657c-3p | TaDES1-5 | 4.5 | -1 | 1 | 21 | 2265 | 2285 | CGUGCUUCCUCGUCGAACGGU | : ::.::.:::: .:::.: | GACCUUUGAUGAGGUGGCAUG | Cleavage | 2 |
| tae-miR9661-5p | TaDES1-12 | 4.5 | -1 | 1 | 21 | 639 | 659 | UGAAGUAGAGCAGGGACCUCA | : :::: ::::::: .::.: | UCAGGUGGCUGCUCUUUUUUA | Cleavage | 1 |
| tae-miR9673-5p | TaDES1-17 | 4.5 | -1 | 1 | 20 | 2607 | 2626 | UAAGAAGCAAAUAGCACAUG | ::: :.:::: ::::.:: | AAUGGGUUAUUGGCUUUUUU | Cleavage | 1 |
| tae-miR9773 | TaDES1-9 | 4.5 | -1 | 1 | 24 | 1254 | 1277 | UUUGUUUUUAUGUUAUUUUGUGAA | :::::::.. :...::::. | UGGGGAAAAUAAUGAAGGGACAAG | Translation | 1 |
| tae-miR1117 | TaDES1-7 | 5 | -1 | 1 | 24 | 1316 | 1339 | UAGUACCGGUUCGUGGCACGAACC | : : : :.:.::.::::::. | CACACAUCCGAUGGACUGGUACUG | Cleavage | 1 |
| tae-miR1119 | TaDES1-13 | 5 | -1 | 1 | 24 | 1908 | 1930 | UGGCACGGCGUGAUGCUGAGUCAG | :: .:::: :::..:. :::::: | AUGCUUCAG-AUCGUGUGGUGCCA | Cleavage | 1 |
| tae-miR1122a | TaDES1-4 | 5 | -1 | 1 | 20 | 2170 | 2189 | UAGAUACAUCCGUAUCUAGA | :. : : :.::::::::.:. | UUAACAAAUGGAUGUAUUUG | Cleavage | 1 |
| tae-miR1133 | TaDES1-17 | 5 | -1 | 1 | 22 | 658 | 679 | CAUAUACUCCCUCCGUCCGAAA | ::: ::::::.::. .:: | GCCGGGAAGGAGGGGGUGGGUG | Cleavage | 1 |
| tae-miR1133 | TaDES1-14 | 5 | -1 | 1 | 22 | 616 | 637 | CAUAUACUCCCUCCGUCCGAAA | ::: ::::::.::. .:: | GCCGGGAAGGAGGGGGUGGGUG | Cleavage | 1 |
| tae-miR1133 | TaDES1-8 | 5 | -1 | 1 | 22 | 486 | 507 | CAUAUACUCCCUCCGUCCGAAA | ::: ::::::.::. .:: | UCCGGGAAGGAGGGGGUGGGUG | Cleavage | 1 |
| tae-miR1133 | TaDES1-7 | 5 | -1 | 1 | 22 | 419 | 440 | CAUAUACUCCCUCCGUCCGAAA | ::: ::::::.::. .:: | GCCGGGAAGGAGGGGGUGGGUG | Cleavage | 1 |
| tae-miR1133 | TaDES1-16 | 5 | -1 | 1 | 22 | 423 | 444 | CAUAUACUCCCUCCGUCCGAAA | ::: ::::::.::. .:: | GCCGGGAAGGAGGGGGUGGGUG | Cleavage | 1 |
| tae-miR1134 | TaDES1-1 | 5 | -1 | 1 | 24 | 2080 | 2103 | CAACAACAACAAGAAGAAGAAGAU | :. ::: :::::::: ::: | CAGAUUGUCUACUUGUUGUGGUUU | Cleavage | 1 |
| tae-miR1135 | TaDES1-13 | 5 | -1 | 1 | 24 | 1364 | 1387 | CUGCGACAAGUAAUUCCGAACGGA | .::: ::.::::.:::: | GAAAGGUGGAUAUAUUUGUUGCAG | Cleavage | 1 |
| tae-miR1135 | TaDES1-7 | 5 | -1 | 1 | 24 | 1440 | 1463 | CUGCGACAAGUAAUUCCGAACGGA | .::: ::.::::.:::: | GGAAGGUGGAUAUAUUUGUUGCAG | Cleavage | 1 |
| tae-miR1135 | TaDES1-16 | 5 | -1 | 1 | 24 | 1438 | 1461 | CUGCGACAAGUAAUUCCGAACGGA | .::: ::.::::.:::: | GAAAGGUGGAUAUAUUUGUUGCAG | Cleavage | 1 |
| tae-miR160 | TaDES1-16 | 5 | -1 | 1 | 21 | 1952 | 1972 | UGCCUGGCUCCCUGUAUGCCA | :: ...:::::: ::::: | GUUCAGGUGGGGAGCGAGGCA | Cleavage | 1 |
| tae-miR164 | TaDES1-10 | 5 | -1 | 1 | 21 | 197 | 217 | UGGAGAAGCAGGGCACGUGCA | :: :.::: :::::::: | CCGCCGCGUCCUCCUUCUCCA | Cleavage | 1 |
| tae-miR164 | TaDES1-6 | 5 | -1 | 1 | 21 | 327 | 347 | UGGAGAAGCAGGGCACGUGCA | ...:: .:::::::: .:: | AUUGUGUUUCCUGCUUCGUCA | Cleavage | 1 |
| tae-miR164 | TaDES1-4 | 5 | -1 | 1 | 21 | 342 | 362 | UGGAGAAGCAGGGCACGUGCA | ...:: .:::::::: .:: | AUUGUGUUUCCUGCUUCGUCA | Cleavage | 1 |
| tae-miR2275-3p | TaDES1-6 | 5 | -1 | 1 | 22 | 349 | 370 | UUUGGUUUCCUCCAAUAUCUCG | : :.::: ::::: :::.:: | AGUGGUAUGGGAGGCCACCGAA | Cleavage | 1 |
| tae-miR319 | TaDES1-9 | 5 | -1 | 1 | 21 | 1445 | 1465 | UUGGACUGAAGGGAGCUCCCU | ::::. :..: :::::.:. | CUGGAGUACUUUGCAGUCUAG | Cleavage | 1 |
| tae-miR5049-3p | TaDES1-9 | 5 | -1 | 1 | 21 | 601 | 622 | AAUA-UGGAUCGGAGGGAGUAC | :::::::::::::::. :::: | GUACUCCCUCCGAUCUUCUAUU | Cleavage | 2 |
| tae-miR5049-3p | TaDES1-9 | 5 | -1 | 1 | 21 | 324 | 344 | AAUAUGGAUCGGAGGGAGUAC | : ::.::::. .::::::: | CUUCUUCCUCUAGUCCAUAUA | Translation | 2 |
| tae-miR5049-3p | TaDES1-2 | 5 | -1 | 1 | 21 | 122 | 142 | AAUAUGGAUCGGAGGGAGUAC | : :: ..:.::::::::.: | AUUCUGUUUUCGAUCCAUGUC | Cleavage | 1 |
| tae-miR5049-3p | TaDES1-1 | 5 | -1 | 1 | 21 | 122 | 142 | AAUAUGGAUCGGAGGGAGUAC | : :: ..:.::::::::.: | AUUCUGUUUUCGAUCCAUGUC | Cleavage | 1 |
| tae-miR5049-3p | TaDES1-3 | 5 | -1 | 1 | 21 | 122 | 142 | AAUAUGGAUCGGAGGGAGUAC | : :: ..:.::::::::.: | AUUCUGUUUUCGAUCCAUGUC | Cleavage | 1 |
| tae-miR5049-3p | TaDES1-13 | 5 | -1 | 1 | 21 | 1054 | 1074 | AAUAUGGAUCGGAGGGAGUAC | : :.::: .:::...:::: | CCAAUUCCUAUGAUUUGUAUU | Cleavage | 1 |
| tae-miR6197-5p | TaDES1-6 | 5 | -1 | 1 | 21 | 2598 | 2618 | UCUGUAAACAAAUGUAGGACG | :.::.. : :::::.:::: | UAUUCUGUUUCUGUUUGCAGA | Translation | 1 |
| tae-miR6197-5p | TaDES1-4 | 5 | -1 | 1 | 21 | 2319 | 2339 | UCUGUAAACAAAUGUAGGACG | :.::.. : :::::.:::: | UAUUCUGUUUAUGUUUGCAGA | Translation | 1 |
| tae-miR6197-5p | TaDES1-17 | 5 | -1 | 1 | 21 | 1968 | 1987 | UCUGUAAACAAAUGUAGGACG | : ::. :.::::::: ::::: | CAUCUAAUAUUUGUU-ACAGA | Cleavage | 1 |
| tae-miR6197-5p | TaDES1-8 | 5 | -1 | 1 | 21 | 1728 | 1747 | UCUGUAAACAAAUGUAGGACG | : ::. :.::::::: ::::: | CAUCUAAUAUUUGUU-ACAGA | Cleavage | 1 |
| tae-miR9654a-3p | TaDES1-3 | 5 | -1 | 1 | 22 | 1796 | 1817 | UUCUGAAAGGCUUGAAGCGAAU | ::: ::::.::.: :::::: | UUUCUCUUCGAGUCCAUCAGAA | Cleavage | 1 |
| tae-miR9657a-3p | TaDES1-5 | 5 | -1 | 1 | 21 | 2265 | 2285 | UGUGCUUCCUCGUCGAACGGU | : ::.::.:::: .:::.. | GACCUUUGAUGAGGUGGCAUG | Cleavage | 1 |
| tae-miR9657b-3p | TaDES1-5 | 5 | -1 | 1 | 21 | 2174 | 2194 | CGUGCUUCCUCGUCGAACGGU | : :: ::.:::: .:::.: | GACCUUGGAUGAGGUGGCAUG | Cleavage | 2 |
| tae-miR9657b-5p | TaDES1-17 | 5 | -1 | 1 | 21 | 64 | 84 | UUCGUCGGAGAAGCAUGUUGC | : : ::::::::..::: | AACCCCUCCUUCUCCGGUGAA | Cleavage | 1 |
| tae-miR9657c-3p | TaDES1-5 | 5 | -1 | 1 | 21 | 2174 | 2194 | CGUGCUUCCUCGUCGAACGGU | : :: ::.:::: .:::.: | GACCUUGGAUGAGGUGGCAUG | Cleavage | 2 |
| tae-miR9664-3p | TaDES1-10 | 5 | -1 | 1 | 21 | 2070 | 2089 | UUGCAGUCCUCGAUGUCGUAG | :.:.:::.::: :.::::: | AAAUGGCAUUGAG-AUUGCAA | Cleavage | 1 |
| tae-miR9666a-3p | TaDES1-10 | 5 | -1 | 1 | 22 | 866 | 887 | CGGUAGGGCUGUAUGAUGGCGA | ..::::: :: ::::::: | AUAUUAUCAUCCAUCCCUACCU | Cleavage | 1 |
| tae-miR9667-5p | TaDES1-3 | 5 | -1 | 1 | 21 | 1339 | 1359 | AAAUAUGGCAAACAAUGAAUG | ..::: :::::.:: :::: | UGUUCCCCGUUUGUCACAUUU | Cleavage | 1 |
| tae-miR9668-5p | TaDES1-14 | 5 | -1 | 1 | 21 | 1625 | 1645 | CCAAUGACAAGUAUUUUCGGA | .:.: :::.::::.. ::: | GGUGGACAUAUUUGUUGCUGG | Cleavage | 1 |
| tae-miR9673-5p | TaDES1-16 | 5 | -1 | 1 | 20 | 2602 | 2621 | UAAGAAGCAAAUAGCACAUG | :: ::.: ::::.::.:: | AAUUUGUUUUUUGUUUUUUU | Cleavage | 1 |
| tae-miR9673-5p | TaDES1-7 | 5 | -1 | 1 | 20 | 808 | 827 | UAAGAAGCAAAUAGCACAUG | .::: :.: ::::::::: | UAUGAUGUGUCUGCUUCUUA | Translation | 1 |
| tae-miR9674b-5p | TaDES1-7 | 5 | -1 | 1 | 21 | 602 | 622 | AUAGCAUCAUCCAUCCUACCC | :. :.:::::::: ::.: | AAAUGAGGUGGAUGAUACUGU | Cleavage | 1 |
| tae-miR9773 | TaDES1-15 | 5 | -1 | 1 | 24 | 1160 | 1183 | UUUGUUUUUAUGUUAUUUUGUGAA | : ...::: :::.::::.:::. | UCUGUAAACUAAUAUAAGAACGUU | Cleavage | 1 |
| tae-miR9773 | TaDES1-1 | 5 | -1 | 1 | 24 | 1697 | 1720 | UUUGUUUUUAUGUUAUUUUGUGAA | :: :::: :: : :::..:::: | GACAAAAAAGAAAAAAAAGGCAAA | Translation | 1 |
| tae-miR9773 | TaDES1-13 | 5 | -1 | 1 | 24 | 2 | 25 | UUUGUUUUUAUGUUAUUUUGUGAA | : : ::: :: : ::::::::: | AGCUCCAAAAAAGACAAAAACAAA | Translation | 1 |
| tae-miR9782 | TaDES1-16 | 5 | -1 | 1 | 24 | 1627 | 1650 | GUAUUAGGUUGGUCAAAUUGACGA | .:::: :::: : :::::: | CAUAUAAUUAAACCAUCAUAAUAC | Cleavage | 1 |

**Supplementary Table S3** Predicted gene specific SSR markers in genomic sequences of TaDES1 members.

| ID | SSR nr. | SSR type | SSR | size | start | end |
| --- | --- | --- | --- | --- | --- | --- |
| TaDES1-2 | 1 | p1 | (A)11 | 11 | 1795 | 1805 |
| TaDES1-8 | 1 | p1 | (T)10 | 10 | 1851 | 1860 |
| TaDES1-8 | 2 | p1 | (T)10 | 10 | 2295 | 2304 |
| TaDES1-10 | 1 | p1 | (A)10 | 10 | 3042 | 3051 |
| TaDES1-14 | 1 | p1 | (T)13 | 13 | 2055 | 2067 |
| TaDES1-17 | 1 | p1 | (T)10 | 10 | 2092 | 2101 |

**Supplementary Table S4** The name of the three key *TaDES1* genes and their homologs across ten wheat cultivars.

| **Three key *TaDES1* genes and their homologs across ten wheat cultivars** | | | |
| --- | --- | --- | --- |
| **Wheat Cultivar** | ***TaDES1-9*** | ***TaDES1-10*** | ***TaDES1-12*** |
| **CM42** | *CM425A387500.1* | *CM425B426600.1* | *CM426A256800.1* |
| **AMN** | [*AMN5A391300.1*](https://wvpn.ustc.edu.cn/http/77726476706e69737468656265737421e7ff449d333f65597d1bc7bf9c54367b4c12ab6735c1/cgi-bin/get_fasta_bedtools.py?database=all_gene&ID=AMN5A391300.1) | [*AMN5B423000.1*](https://wvpn.ustc.edu.cn/http/77726476706e69737468656265737421e7ff449d333f65597d1bc7bf9c54367b4c12ab6735c1/cgi-bin/get_fasta_bedtools.py?database=all_gene&ID=AMN5B423000.1) | [*AMN6B336000.1*](https://wvpn.ustc.edu.cn/http/77726476706e69737468656265737421e7ff449d333f65597d1bc7bf9c54367b4c12ab6735c1/cgi-bin/get_fasta_bedtools.py?database=all_gene&ID=AMN6B336000.1) |
| **BJ8** | [*BJ85A407400.1*](https://wvpn.ustc.edu.cn/http/77726476706e69737468656265737421e7ff449d333f65597d1bc7bf9c54367b4c12ab6735c1/cgi-bin/get_fasta_bedtools.py?database=all_gene&ID=BJ85A407400.1) | [*BJ85B444700.1*](https://wvpn.ustc.edu.cn/http/77726476706e69737468656265737421e7ff449d333f65597d1bc7bf9c54367b4c12ab6735c1/cgi-bin/get_fasta_bedtools.py?database=all_gene&ID=BJ85B444700.1) | [*BJ86A249400.1*](https://wvpn.ustc.edu.cn/http/77726476706e69737468656265737421e7ff449d333f65597d1bc7bf9c54367b4c12ab6735c1/cgi-bin/get_fasta_bedtools.py?database=all_gene&ID=BJ86A249400.1) |
| **ZM22** | [*HD61725A369300.1*](https://wvpn.ustc.edu.cn/http/77726476706e69737468656265737421e7ff449d333f65597d1bc7bf9c54367b4c12ab6735c1/cgi-bin/get_fasta_bedtools.py?database=all_gene&ID=HD61725A369300.1) | [*HD61725B437000.1*](https://wvpn.ustc.edu.cn/http/77726476706e69737468656265737421e7ff449d333f65597d1bc7bf9c54367b4c12ab6735c1/cgi-bin/get_fasta_bedtools.py?database=all_gene&ID=HD61725B437000.1) | [*HD61726B327700.1*](https://wvpn.ustc.edu.cn/http/77726476706e69737468656265737421e7ff449d333f65597d1bc7bf9c54367b4c12ab6735c1/cgi-bin/get_fasta_bedtools.py?database=all_gene&ID=HD61726B327700.1) |
| **ZM22** | *ZM225A395800.1* | *ZM225B426300.1* | *ZM226A251300.1* |
| **XN6028** | *XN60285A389900.1* | *XN60285B427000.1* | *XN60286B319600.1* |
| **MZM** | [*MZM5A392600.1*](https://wvpn.ustc.edu.cn/http/77726476706e69737468656265737421e7ff449d333f65597d1bc7bf9c54367b4c12ab6735c1/cgi-bin/get_fasta_bedtools.py?database=all_gene&ID=MZM5A392600.1) | [*MZM5B432300.1*](https://wvpn.ustc.edu.cn/http/77726476706e69737468656265737421e7ff449d333f65597d1bc7bf9c54367b4c12ab6735c1/cgi-bin/get_fasta_bedtools.py?database=all_gene&ID=MZM5B432300.1) | [*MZM6B326100.1*](https://wvpn.ustc.edu.cn/http/77726476706e69737468656265737421e7ff449d333f65597d1bc7bf9c54367b4c12ab6735c1/cgi-bin/get_fasta_bedtools.py?database=all_gene&ID=MZM6B326100.1) |
| **NC4** | [*NC45A391800.1*](https://wvpn.ustc.edu.cn/http/77726476706e69737468656265737421e7ff449d333f65597d1bc7bf9c54367b4c12ab6735c1/cgi-bin/get_fasta_bedtools.py?database=all_gene&ID=NC45A391800.1) | [*NC45B423800.1*](https://wvpn.ustc.edu.cn/http/77726476706e69737468656265737421e7ff449d333f65597d1bc7bf9c54367b4c12ab6735c1/cgi-bin/get_fasta_bedtools.py?database=all_gene&ID=NC45B423800.1) | [*NC46A254500.1*](https://wvpn.ustc.edu.cn/http/77726476706e69737468656265737421e7ff449d333f65597d1bc7bf9c54367b4c12ab6735c1/cgi-bin/get_fasta_bedtools.py?database=all_gene&ID=NC46A254500.1) |
| **XY6** | [*XY65A392600.1*](https://wvpn.ustc.edu.cn/http/77726476706e69737468656265737421e7ff449d333f65597d1bc7bf9c54367b4c12ab6735c1/cgi-bin/get_fasta_bedtools.py?database=all_gene&ID=XY65A392600.1) | [*XY65B427700.1*](https://wvpn.ustc.edu.cn/http/77726476706e69737468656265737421e7ff449d333f65597d1bc7bf9c54367b4c12ab6735c1/cgi-bin/get_fasta_bedtools.py?database=all_gene&ID=XY65B427700.1) | [*XY66A261400.1*](https://wvpn.ustc.edu.cn/http/77726476706e69737468656265737421e7ff449d333f65597d1bc7bf9c54367b4c12ab6735c1/cgi-bin/get_fasta_bedtools.py?database=all_gene&ID=XY66A261400.1) |
| **YM158** | [*YM1585A409100.1*](https://wvpn.ustc.edu.cn/http/77726476706e69737468656265737421e7ff449d333f65597d1bc7bf9c54367b4c12ab6735c1/cgi-bin/get_fasta_bedtools.py?database=all_gene&ID=YM1585A409100.1) | [*YM1585B425300.1*](https://wvpn.ustc.edu.cn/http/77726476706e69737468656265737421e7ff449d333f65597d1bc7bf9c54367b4c12ab6735c1/cgi-bin/get_fasta_bedtools.py?database=all_gene&ID=YM1585B425300.1) | [*YM1586A262100.1*](https://wvpn.ustc.edu.cn/http/77726476706e69737468656265737421e7ff449d333f65597d1bc7bf9c54367b4c12ab6735c1/cgi-bin/get_fasta_bedtools.py?database=all_gene&ID=YM1586A262100.1) |

**Supplementary Table S5** Variations in three key *TaDES1* genes and their homologs across ten wheat cultivars.

| **Variations in three key TaDES1 genes and their homologs across ten wheat cultivars** | | | |
| --- | --- | --- | --- |
| **Wheat Cultivar** | **TaDES1-9** | **TaDES1-10** | **TaDES1-12** |
| **CM42** | Substitution | Substitution | Substitution |
| **AMN** | Substitution | Substitution | - |
| **BJ8** | - | Substitution | Substitution |
| **ZM22** | - | Substitution | Substitution |
| **ZM22** | - | Substitution | Substitution |
| **XN6028** | Substitution | Substitution | - |
| **MZM** | Substitution | Substitution | - |
| **NC4** | - | Substitution | Substitution |
| **XY6** | Substitution | Substitution | Substitution |
| **YM158** | Substitution | - | Substitution |
| **Identity** | 99.95% | 99.95% | 99.11% |

**Supplementary Table S6** Primer sequences used for qPCR.

| **Gene name** | **Primer ID** | **Primer Sequence** |
| --- | --- | --- |
| TaGAPDH | TaGAPDH-F | CCACTAACTGCCTTGCTCCTCTTG |
|  | TaGAPDH-R | CTTCCACCTCTCCAGTCCTTGCT |
| TaDES1-9 | TaDES1-9F | ATGCCATCATACACCAGCCT |
|  | TaDES1-9R | GTGTCCTCCCATATCTCCGG |
| TaDES1-10 | TaDES1-10F | ATGAGGCTGGTGAGGAACAA |
|  | TaDES1-10R | TGTCCCTGATGTTGAGCACT |
| TaDES1-12 | TaDES1-12F | TTGCCACCAACATCACACAG |
|  | TaDES1-12R | TCAAGCCTTTCTCCTCTGCA |
