# Supplementary material for: Genome‐Wide Identification and Expression Analysis of TaDES1 Gene Family Responded to Biotic and Abiotic Stress in Wheat ( Triticum aestivum L.)
Source: Food Sci Nutr. 2025 Jul 8;13(7):e70504. doi: 10.1002/fsn3.70504 (PMC12235670; doi:10.1002/fsn3.70504)
Supplement: Supplementary file 3 — Data S1. Deduced amino acid sequences of DES1 analyzed in this study. [file FSN3-13-e70504-s003.docx]

**Supplementary text 1** **Deduced amino acid sequences of *DES1* genes analyzed in this study.**

>AtOAS-TLA

MASRIAKDVTELIGNTPLVYLNNVAEGCVGRVAAKLEMMEPCSSVKDRIGFSMISDAEKKGLIKPGESVL

IEPTSGNTGVGLAFTAAAKGYKLIITMPASMSTERRIILLAFGVELVLTDPAKGMKGAIAKAEEILAKTP

NGYMLQQFENPANPKIHYETTGPEIWKGTGGKIDGFVSGIGTGGTITGAGKYLKEQNANVKLYGVEPVES

AILSGGKPGPHKIQGIGAGFIPSVLNVDLIDEVVQVSSDESIDMARQLALKEGLLVGISSGAAAAAAIKL

AQRPENAGKLFVAIFPSFGERYLSTVLFDATRKEAEAMTFEA

>AtOAS-TLB

MAATSSSAFLLNPLTSRHRPFKYSPELSSLSLSSRKAAAFDVSSAAFTLKRQSRSDVVCKAVSIKPEAGV

EGLNIADNAAQLIGKTPMVYLNNVVKGCVASVAAKLEIMEPCCSVKDRIGYSMITDAEEKGLITPGKSVL

VESTSGNTGIGLAFIAASKGYKLILTMPASMSLERRVLLRAFGAELVLTEPAKGMTGAIQKAEEILKKTP

NSYMLQQFDNPANPKIHYETTGPEIWEDTRGKIDILVAGIGTGGTITGVGRFIKERKPELKVIGVEPTES

AILSGGKPGPHKIQGIGAGFVPKNLDLAIVDEYIAISSEEAIETSKQLALQEGLLVGISSGAAAAAAIQV

AKRPENAGKLIAVVFPSFGERYLSTQLFQSIREECEQMQPEL

>AtOAS-TLC

MVAMIMASRFNREAKLASQILSTLLGNRSCYTSMAATSSSALLLNPLTSSSSSSTLRRFRCSPEISSLSF

SSASDFSLAMKRQSRSFADGSERDPSVVCEAVKRETGPDGLNIADNVSQLIGKTPMVYLNSIAKGCVANI

AAKLEIMEPCCSVKDRIGYSMVTDAEQKGFISPGKSVLVEPTSGNTGIGLAFIAASRGYRLILTMPASMS

MERRVLLKAFGAELVLTDPAKGMTGAVQKAEEILKNTPDAYMLQQFDNPANPKIHYETTGPEIWDDTKGK

VDIFVAGIGTGGTITGVGRFIKEKNPKTQVIGVEPTESDILSGGKPGPHKIQGIGAGFIPKNLDQKIMDE

VIAISSEEAIETAKQLALKEGLMVGISSGAAAAAAIKVAKRPENAGKLIAVVFPSFGERYLSTPLFQSIR

EEVEKMQPEV

>AtDES1

MEDRVLIKNDVTELIGNTPMVYLNKIVDGCVARIAAKLEMMEPCSSIKDRIAYSMIKDAEDKGLITPGKS

TLIEATGGNTGIGLASIGASRGYKVILLMPSTMSLERRIILRALGAEVHLTDISIGIKGQLEKAKEILSK

TPGGYIPHQFINPENPEIHYRTTGPEIWRDSAGKVDILVAGVGTGGTVTGTGKFLKEKNKDIKVCVVEPS

ESAVLSGGKPGPHLIQGIGSGEIPANLDLSIVDEIIQVTGEEAIETTKLLAIKEGLLVGISSGASAAAAL

KVAKRPENVGKLIVVIFPSGGERYLSTELFESVRYEAENLPVE

>BnDES1

MEERYMIKKDVTELIGYTPMVYLNRIVDGCVARIAAKLEMMQPCSSVKDRIAYSMIKDAEDKGLIKPGES

TLIEPTAGNTGIGLACIGAARGYKVTLLMPSTMSLERRIILKVLGAELHLTDMSIGIKGLLEKTEEMLNK

TPGGFVPQQFENLANPEIHYQTTGPEIWRDSAGKVDIFIAGVGTGGTVTGVGRFLKEMNKDIKVIAVEPT

ESPVLSGGEPGRHLIQGIGAGIIPANLDLSIVDEIIQVTGEEAIETAKLLALKEGLLVGISSGAAAAAAL

KVAKRPENAGKLIAVLFPSGGERYLSTKLFDSVRFEAENLPVE

>SiDES1

MASLVRRRFYSSEASFVQRLRDLPKYLPGTKIKTQVSQLIGKTPLVYLNKVSEGCGAYIAVKQEMMQPTS

SIKDRPAFAMINDAEKKGLITPGKTTLIEPTSGNMGISMAFMAAMKGYKMILTMPSYTSLERRVTMRAFG

ADLVITDPTKGMGGTIKKAYDLLESTPNAYMLQQFSNPANTQVHFETTGPEIWEDTQGNVDIFVMGIGSG

GTVSGVGQYLKSKNPNVKIYGIEPAESNVLNGGKPGPHEITGNGVGFKPDILDMDVMEEVLMVSSEESVN

MARELALKEGLMVGISSGANTVAALRLANRPENKGKLIVTIHPSFGERYLSSVLYEDLRKEAQNMQPVSV

D

>TaDES1-1

MRQWCPNETIAKDVTVLIGRTPLVYLNKVVTGCEARIAAKLEIMGPCSSIKDRIGYSMIT

DAEEKGLITPGKSVLIEPTGGNTGIGLAFMAAAKGYKLIVTMPSSVSTERRTVLKAFGAE

VVLTDPLRAMDDVVRRAEDIAAKTPNSYVLQQFENPANTKVHYETTGPEIWSGTAGAVDI

LVAGIGTGGTITGAGKYLKEMNPEIQIYGVEPSECAVLSGGKPGPHKIQGLGAGFVPGVL

DVSILDEVFQITNEEAASMARQIALKEGLLVGMSSGATAAAAIRVARRVENRGKLIVVVF

ASCGERYLSSFLFESIKKEAENMVLEP

>TaDES1-2

MGQWCPSETIAKDVTELIGRTPLVYLNKVVTGCEARVAAKLEIMGPCSSIKDRIGYSMIA

DAEEKGLITPGKSVLIEPTGGNTGIGLAFMAAAKGYKLIVTMPSSVSTERRTVLKAFGAE

VVLTDPLRAMDDVVRRAEEIAAKTPNSYVLQQFENPANTKVHYETTGPEIWSGTAGAVDI

LVAGIGTGGTITGAGKYLKEMNPEIQIYGVEPSECAVLSGGKPGPHKIQGLGAGFVPGVL

DVSILDEVFQITNEEAASMARQIALKEGLLVGMSSGATAAAAIRVARRVENRGKLIVVVF

ASCGERYLSSFLFESIKKEAENMVLEP

>TaDES1-3

MGQWCPSETIAKDVTELIGRTPLVYLNKVVTGCEARVAAKLEIMGPCSSIKDRIGYSMIA

DAEEKGLITPGKSVLIEPTGGNTGIGLAFMAAAKGYKLIVTMPSSVSTERRTVLKAFGAE

VVLTDPLRAMDDVVRRAEEIAARTPNSYVLQQFENPANTKVHYETTGPEIWSGTAGAVDI

LVAGIGTGGTITGAGKYLKEMNPEIQIYGVEPSESAVLSGGKPGPHKIQGLGAGFVPGVL

DVSILDEVFQITNEEAASMARQIALKEGLLVGMSSGATAVAAIRVARRVENRGKLIVVVF

ASCGERYLSSFLFESIRNEAENMVFEP

>TaDES1-4

MASPPACSLLLPAIPSAPAYASVPRAPRFLTCPRAVTAHRPLPTASSPKVAAPAAIEIPE

EYADDVEAVNIAVDVTQLIGKTPMVYLNNVVEGCVANIAAKLEYMGPCRSVKDRIALSMI

SDAEEKGLISPNKTILVEPTTGNTGIGLAAVAAARGYKMIATMPSSIDVERRILVRAFGA

DIVLTDPTTGLKGAVDKAEEIVSKTPNAYMFQQFNNSANSEIHFQTTGPEIWEDTLGTVD

ILVASIGTGDTITGTGRYLKMMNRDIKVIGVEPAETSVISGDKPGYIPSILDVQLLDEVT

TAEAVDVARLLALKEGLLVGISSGAAAIAAINVAKRPENAGKLIAVIFPSFGERYISSIL

FRPIYNSVRRMRKR

>TaDES1-5

MASPPACSLLLPAIPSAPAYASVPRAPRFLTCPRAVTAHRPFPTASSPKVAAPAAVEIPE

EYVDDVEAVNIAVDVTQLIGKTPMVYLNNVVEGCVANIAAKLEYMGPCRSVKDRIALSMI

SDAEDKGLISPNKTILVEPTTGNTGIGLAAVAAARGYKMIATMPSSIDVERRILVRAFGA

DIVLTDPTTGLKGAVDKAEEIVSKTPNAYMFQQFNNSANSEIHFQTTGPEIWEDTLGTVD

ILIASIGTGGTITGTGRYLKMMNRDIKVIGVEPAETSVISGDKPGYIPSILDVQLLDEVT

TAEAVDVARLLALKEGLLVGISSGAAAIAAINVAKRPENAGKLIAVIFPSFGERYISSIL

FRPIYNSVRRMRKR

>TaDES1-6

MAMASPPACSLLLPAISSAPAYASVPRAPRFLTCPRAVTAHRPLPTASSPKVAAPAAVEI

PEEYADDVEAVNIAVDVTQLIGKTPMVYLNNVVEGCVANIAAKLEYMGPCRSVKDRIALS

MISDAEEKGLISPNKTILVEPTTGNTGIGLAAVAAARGYKMIATMPSSIDVERRILVRAF

GADIVLTDPTIGLKGAVDKAEEIVSKTPNAYMFQQFNNPANSEIHFQTTGPEIWEDTLGT

VDILVASIGTGGTITGTGRYLKMMNRDIKVIGVEPAETSVISGDKPGYIPSILDVQLLDE

VVKVTTAEAVDVARLLALKEGLLVGISSGAAAIAAINVAKRPENAGKLIAVIFPSFGERY

ISSILFRPIYNSVRRMRKR

>TaDES1-7

MAPGAEEAEGAGRRGVPSLLAGGGTGQEEHIASDVTQLIGWTPLIELKRIAGKEGVGARI

VGKIEAYQPLCSVKDRSALRMIEDAEEKGLISPGVTTLVEPTSGNLGLGLVLIALSKGYR

FVAVMPGQYSLDKQILLRYMGAELFITDPALGFPGQVEKVEQLKKELPNVHVLDQFSNPA

NPEAHIRWTGPEIWKDTAGKVDIFVAGSGSGGTVSGVGKYLKTQNPNVKIICVEPAESPV

ISGGERGKHEIQGIGPGFLPEILDTLVIDEVLTVTTEEAMVNARRLAMEEGLLVGISSGA

NLAACLKVAAREENKGKMIVTMFPSGGERYMNSDLFAAVREECIAMTF

>TaDES1-8

MATGDGAEGTGRRGVPSLLADGGTGQEEHIASDVTQLIGWTPLIELKRISGKEGVGARIV

GKIEAYQPLCSVKDRSALRMIEDAEEKGLISPGVTTLVEPTSGNLGLGLVLVALSKGYRF

VAVMPGQYSLDKQILLRYMGAELFITDPTLGFPGLVDKVEQLKKELPNVHVLDQFANPAN

PDAHIRWTGPEIWKDTAGKVDIFVAGSGSGGTVSGVGKYLKMQNPNVKIICVEPAESPVI

SGGEPGKHKIQGIGPGFLPEILDTSIIDETVTVTTEDAMTNARRLAMEEGLLVGISSGAN

LAACLKVAAREENKGKMIVTMFPSGGERYMNSDLFAAVREECIAMTF

>TaDES1-9

MERMVMRLMRNKNQSLRQLQGAAAGGLASSSSAPSTAGAAASPFSTLQQQEDHPAPGVLN

IRDTAAHLIGRTPLVYLNRVTEGCGARVAAKLEFLQPSFSVKDRPAISMIEDAEKKGLIT

PGKTTLIEPTSGNMGIGLAFMAALKGYELVLTMPSYTSLERRVVMKAFGAQLVLTDPAKG

MGGTVRKATQLYENHPSAFMLQQFENPANVQVHYETTGPEIWEDTLGQVDIFVMGIGSGG

TVTGVGKYLKEKNPNAKIYGVEPAEANVLNGGKPGPHLITGNGVGFKPDILDMDIMEKVL

EVKGEDAVKMAKQLALQEGLLVGISSGANTVAAIELAKRPENKGKLIVTVHPSAGERYLS

SALFEGLRKEAEAMQPVPVD

>TaDES1-10

MERVVMRLVRNKQSLRQLQGAAAGGLASSSSASSTAGAAASSFSTLQQQQEDHPGVLNIR

DTAAHLIGRTPLVYLNKVTEGCGARVAAKLEFLQPSFSVKDRPAISMIEDAEKKGLITPG

KTTLIEPTSGNMGIGLAFMAALKGYELVLTMPSYTSLERRVVMKAFGAQLVLTDPAKGMG

GTVRKATQLYENHPSAFMLQQFENPANVQVHYETTGPEIWEDTLGQVDIFVMGIGSGGTV

TGVGKYLKEKNPNAKIYGVEPAEANVLNGGKPGPHLITGNGVGFKPDILDMDIMEKVLEV

KSEDAVTMAQQLALQEGLLVGISSGANTVAAIELAKRPENKGKLIVTVHPSAGERYLSSA

LFEGLRKEAEAMQPVPVD

>TaDES1-11

MEPQEGRKGIPSLLSSQGECIATNITQLIGWTPLIELRNITEKDGIGARLIGKIEPYQPL

SSVKDRSALRLIEDAEEKGLITPGITTLLGVTSGNLGIGVAFIAAQKGYKFIALMPAKLS

LDKQILMRFLGVEVVLVDAVQHGFKALLDRVEQMKKDVEDVYVLDQFTNPANPDAHFRWT

GPEIWKDTAGKVDIFIAASGSGGTITGVGRYLKTKNPFVKLICVEPAESPVISGGEPAFH

NILGIGPGFVPEILDRSQIDEIVTVTTQEAMDMARRLAREEGLLVGISSGANAAACLKVA

SREENRGKMIVTMFSSGAERYLNSELFAQVKEECVNINMTF

>TaDES1-12

MEPQEGRKGIPSLLSSQGECIATNITQLIGWTPLIELRNITEKDSIGARLIGKIEPYQPL

SSVKDRSALRLIEDAEEKGLITPGITTLLGVTSGNLGIGVAFIAAQKGYKFIALMPAKLS

LDKQILMRYLGVEVVLVDAVQHGFKALLDRVEQMKKDVEDVYVLDQFTNPANPDAHFRWT

GPEIWKDTAGKVDIFIAASGSGGTITGVGRYLKTKNPSVKLICVEPAESPVISGGEPAFH

NILGIGPGFVPEILDRSQIDEIVTVTTQEAMDMARRLAREEGLLVGISSGANAAACLKVA

SREENRGKMIVTMFSSGAERYLNSELFAQVKEECVNVNMTF

>TaDES1-13

MAPGAEEAEGAGRRGVPSLLAGGGTGQEEHIATDVTQLIGWTPLIELKRIAGKEGVGARI

VGKIEAYQPLCSVKDRGALRMIEDAEEKGLISPGVTTLVEPTSGNLGLGLVLIALSKGYR

FVAVMPGQYSLDKQILLRYMGAELFITDPALGFPGQVEKVEQLKKELPNVHVLDQFSNPA

NPEAHIRWTGPEIWKDTAGKVDIFVAGSGSGGTVSGVGKYLKMQNPNVKIICVEPAESPV

ISGGERGKHKIQGIGPGFVPEILDSSIIDEVLTVTTEEAMVNARRLAMEEGLLVGISSGA

NLAACLKVAVREENKGKMIVTMFPSGGERYMNSDLFAAVREECIAMTF

>TaDES1-14

MATGEGADGTGRRGVPSLLQDGGTGQDEHIASDVTQLIGWTPLIELRRIAGKEGVGARIV

GKIEAYQPLCSVKDRSALRMIEDAEEKGLISPGVTTLVEPTSGNLGLGLVLIALSKGYRF

VAVMPGQYSLDKQILLRYMGAELFITDPTLGFPGITDKVEQLKKELPNVHVLDQFSNPAN

PDAHIRMTGPEIWKDTAGKVDIFVAGSGSGGTVSGVGKYLKMQNPNVKIICVEPTESSVI

SGGEPGKHKIQGIGPGFLPEILDTSVIDETVTVTTEDAMVNARRLAMEEGLLVGISSGAN

LAACLKVAAREENKGKMIVTMFPSGGERYMNSDLFAAVREECIAMTF

>TaDES1-15

MAVEEEGRKGIPSLLSSGDGEENIASNITQLIGWTPLIEMKNIAKKDGVEARLVGKMEAY

QPLCSVKDRSALRMIEDAEEKGLISPGVTTLIEPTSGNQGIGMVFIAIQKGYRFIAVMPA

KYSLDKQMLLRFLGAELILTDPANGFKGMIGKVEELMKTIPNSHCLNQVTNPANPDAHFK

WTGPEIWKDTAGKVDMFVAAVGSGGTLTGVGKYLKMKNPSIKIVCVEPSESAVISGGSPG

SHKIQGTGPGFIPEVLDTSVIDEVVTVSTEEAMVMARRLAREEGLLVGISSGANVAACIK

IAAREGNEGKMIVTIFPSAGERYMNSDLFALVREECENMTF

>TaDES1-16

MGRSWGAQPLKLQKNTKTNSNLTTCRADSSAVAALATLSLLQREEHPSMPPGAEEAERAG

RRGVPSLLAGGGTGQEEHIASDVTQLIGWTPLIELKRIAGKEGVGARIVGKIEAYQPLCS

VKDRSALRMIEDAEEKGLISPGVTTLVEPTSGNLGLGLVLIALSKGYRFVAVMPGQYSLD

KQILLRYMGAELFITDPALGFPGQVEKVEQLKKELPNVHVLDQFSNPANPEAHIRWTGPE

IWKDTAGKVDIFVAGSGSGGTVSGVGKYLKMQNPNVKIICVEPAESPVISGGERGKHKIQ

GIGPGFVPEILDSSVIDEVLTVTTEEAMVNARRLAMEEGLLVGISSGGNLAACLKVAARE

ENKGKMIVTMFPSGGERYMNSDLFAAVREECIAMTF

>TaDES1-17

MATGEGADGTGRRGVPSLLADGGTGQEEHIASDVTQLIGWTPLIELKRIAGKEGVGARIV

GKIEAYQPLCSVKDRSALRMIEDAEEKGLISPGVTTLVEPTSGNLGLGLVLIALSKGYRF

VAVMPGQYSLDKQILLRYMGAELFITDPTLGFPGITDKVEQLKKELPNVHVLDQFSNPAN

PDAHIRWTGPEIWKDTAGKVDIFVAGSGSGGTVSGVGKYLKMQNPNVKIICVEPAESPVI

SGGEPGKHKIQGIGPGFLPEILDTSVIDETVTVTTEDAMTNARRLAMEEGLLVGISSGAN

LAACLKVAAREENKGKMIVTMFPSGGERYMNSDLFATVREECIAMTF

>TaDES1-18

MAVEEEGRKGIPSLLSSGEENIASNITQLIGWTPLIEMKNIAKKDGVEARLVGKMEAYQP

LCSVKDRSALRMIEDAEEKGLISPGVTTLIEPTSGNQGIGMVFIAVQKGYRFIAVMPAKY

SLDKQMLLRFLGAELILTDPANGFKGMIGKVEELMKTIPNSYCLNQVTNPANPDAHFKWT

GPEIWKDTAGKVDMFVAAVGSGGTLTGVGKYLKMKNPSIKLVCVEPSESAVISGGSPGSH

KIQGTGPGFIPEVLDTSVIDEVVTVSTEEAMTMARRLAREEGLLVGISSGANVAACIKIA

MREENQGKMIVTIFPSAGERYMNSDLFAVVRGECENMTF
